# Bacteriophage-encoded 24B_1 molecule resembles herpesviral microRNAs and plays a crucial role in the development of both the virus and its host

**DOI:** 10.1371/journal.pone.0296038

**Published:** 2023-12-20

**Authors:** Sylwia Bloch, Natalia Lewandowska, Joanna Zwolenkiewicz, Paulina Mach, Aleksandra Łukasiak, Mikołaj Olejniczak, Logan W. Donaldson, Grzegorz Węgrzyn, Bożena Nejman-Faleńczyk

**Affiliations:** 1 Department of Molecular Biology, University of Gdansk, Gdansk, Poland; 2 Institute of Molecular Biology and Biotechnology, Adam Mickiewicz University in Poznan, Poznan, Poland; 3 Department of Biology, York University, Toronto, Canada; University of Pennsylvania Perelman School of Medicine, UNITED STATES

## Abstract

The 24B_1 small non-coding RNA molecule has been identified in *Escherichia coli* after induction of Shiga toxin-converting bacteriophage Φ24_B_. In this work, we focused on its direct role during phage and bacterial host development. We observed that in many aspects, this phage sRNA resembles herpesviral microRNAs. Similar to microRNAs, the mature 24B_1 is a short molecule, consisting of just 20 nucleotides. It is generated by cleaving the 80-nt long precursor transcript, and likely it undergoes a multi-step maturation process in which the Hfq protein plays an important role, as confirmed by demonstration of its binding to the 24B_1 precursor, but not to the 24B_1 mature form. Moreover, 24B_1 plays a significant role in maintaining the prophage state and reprogramming the host’s energy metabolism. We proved that overproduction of this molecule causes the opposite physiological effects to the mutant devoid of the *24B_1* gene, and thus, favors the lysogenic pathway. Furthermore, the 24B_1 overrepresentation significantly increases the efficiency of expression of phage genes coding for proteins CI, CII, and CIII which are engaged in the maintenance of the prophage. It seems that through binding to mRNA of the *sdhB* gene, coding for the succinate dehydrogenase subunit, the 24B_1 alters the central carbon metabolism and causes a drop in the ATP intracellular level. Interestingly, a similar effect, called the Warburg switch, is caused by herpesviral microRNAs and it is observed in cancer cells. The advantage of the Warburg effect is still unclear, however, it was proposed that the metabolism of cancer cells, and all rapidly dividing cells, is adopted to convert nutrients such as glucose and glutamine faster and more efficiently into biomass. The availability of essential building blocks, such as nucleotides, amino acids, and lipids, is crucial for effective cell proliferation which in turn is essential for the prophage and its host to stay in the lysogenic state.

## Introduction

Shiga toxin-producing *Escherichia coli* (STEC) bacteria are highly harmful to humans as they contain lambdoid prophages (called Stx phages) bearing the *stx* genes coding for Shiga toxins, the major agents responsible for the development of severe diseases. Stx phages bear many similarities in the life cycle and genomic organization to bacteriophage λ, the most-investigated member of this family [1, 2]. The Stx phage lifecycle begins when the phage adsorbs to the surface of the host bacteria and injects its linear double-stranded DNA into the cell. At this stage, depending on the intracellular conditions, phage may develop according to one of two alternative pathways, lysogenic or lytic [3, 4]. When the lysogenic pathway is chosen, phage DNA is incorporated into *E*. *coli* genome (forming a prophage), replicated together with the bacterial genome, and then transmitted to daughter cells during cell division. The excision of the phage genome occurs following prophage induction. This may be caused by different factors like UV, antibiotics, or hydrogen peroxide that is naturally produced in the human body [5–9]. As a consequence, viral DNA starts to replicate separately from the host bacterial chromosome as an extrachromosomal element [10, 11]. This leads to the synthesis of phage structural and lysis proteins. In effect, the assembly of multiple new phage particles, disruption of the bacterial host cell, and release of large amounts of Shiga toxins and Stx progeny phages occur [12, 13].

The progressive production of Shiga toxins causes bloody diarrhea as the first symptom of human infection. Other symptoms like abdominal pain and vomiting may also occur. Symptoms of food poisoning maintain around 10 days, however, 15–20% of patients infected with these pathogens progress to severe complications such as hemolytic uremic syndrome, thrombocytopenia, or hemorrhagic colitis. Children under five years old and the elderly are most susceptible to severe complications with mortality as high as 87% [14–16]. The main problem of human infection by STEC is a lack of known effective treatment strategies. There is serious doubt of using antibiotics, as they are prophage inducers that indirectly stimulate the expression of genes coding for Shiga toxins and consequently, enhance the severity of disease symptoms [6, 16–18].

The severity of the infection and the development of complications depend on the amount of Shiga toxin produced by bacteria. Importantly, the expression of *stx* genes is tightly linked to lytic development which starts after prophage induction. Otherwise, during lysogeny, the majority of phage genes (including these coding for Shiga toxins) are suppressed. In general, the switch between lysogenic and lytic pathway is based on the activation and inactivation of the major phage repressor of the lytic development (called CI in the case of lambdoid phages). This DNA-binding repressor is involved in the inhibition of the transcription from the main phage promoters *p*_L_ and *p*_R_. The binding of the repressor to the operator sites is a well-known mechanism of negative regulation that inhibits transcriptional initiation and thus suppresses the expression of the genes essential for the lytic cycle, synthesis of toxins, and phage lytic development at all. Before a phage enters the lytic pathway, repressors must be inactivated [19]. The best known mechanism responsible for the inactivation of the phage repressors in lambdoid phages involves the host SOS system that can be induced by physiological changes in the host cells, UV light irradiation, hydrogen peroxide treatment, or antibiotics. In most cases, the inducers of the SOS response cause DNA damage which results in the activation of the RecA protein and subsequent degradation of both the bacterial LexA protein and the phage-encoded CI repressor. The second mechanism to control the repressor activity uses phage-encoded antirepressor protein (frequently called Ant) which acts through the competitive binding with the repressor to the same operator-binding site. Some of the identified antirepressor genes are under the direct control of the LexA, however, some reports indicate that also small non-coding RNAs may be involved in this regulation. For example, the Ant antirepressor synthesis is regulated during the development of bacteriophage P22 by the small antisense RNA named Sar. Notably, this sRNA molecule acts in *trans* by binding to the ribosome binding site of the Ant mRNA and inhibits Ant production at a post-transcriptional level [20, 21]. Similarly, in phage N15 which is a temperate virus of *E*. *coli* related to lambdoid phages, the antirepressor gene is negatively regulated by a small RNA, named CA [22].

Recently, our group discovered 24B_1, a 20-nt microRNA-like molecule, after induction of Shiga toxin-converting bacteriophage Φ24_B_ [23]. Importantly this molecule has been shown to stimulate the lysogenic pathway of phage lifecycle in *E*. *coli* bacteria. We speculated that 24B_1 may act as a regulator of D_ant antirepressor gene expression and thus indirectly control the activity of the repressor of the phage lytic cycle. Previously, we have tested aspects of the development of bacteriophage Φ24_B_ bearing a deletion of the sequence encoding the 24B_1 precursor molecule, that is located between genes *lom* and *vb_24B_43* of the Φ24_B_ phage genome. The obtained results indicated that the lytic developmental pathway was more efficient in the mutant phage lacking the 24B_1 molecule [23]. These promising results prompted us to take a closer look at the 24B_1 role during the phage development in bacteria. In this report, we decided to analyze the influence of the overproduced 24B_1 molecule on the development of the phage as well as its host. Manipulation of the phage lysogenic-lytic decision through microRNA-like particles offers a new way to understand developmental pathways and identify possible therapeutic interventions against pathogenic *E*. *coli* infections.

## Materials and methods

### Bacterial strains, bacteriophages, and plasmids

*E*. *coli* MG1655 strain and its derivatives employed in this work, are presented in Table 1. The deletion mutant, lacking the sequence of the small, microRNA-size, 24B_1 molecule was constructed as described previously by [23] by using *E*. *coli* MG1655 (Φ24_B_) strain and the Quick and Easy *E*. *coli* Gene Deletion Kit (Gene Bridges, Heidelberg, Germany). Phages Φ24_B_ [24] and Φ24_B_Δ24B_1 [23] were from the collection of the Department of Molecular Biology of the University of Gdansk (Poland). Plasmid pUC18 (Thermo Fisher Scientific Inc., Waltham, MA, USA) was used as a control variant in all experiments presented in this work. Plasmid pUC18_24B_1, bearing the 189-nt region of Φ24_B_ genome encompassing the identified 24B_1 sequence, was described by us earlier [23]. The 24B_1 transcript production from pUC18_24B_1 plasmid was also confirmed previously [23]. The plasmid bearing an additional copy of the *24B_1* gene was incorporated into the *E*. *coli* MG1655 strain and used to overproduce the 24B_1 molecule in cells infected with wild-type phage Φ24_B_.

**Table 1:** *E*. *coli* MG1655 strain and its derivatives.

*E*. *coli strains*	Relevant genotype or description	References
MG1655	F^−^λ^–^ *ilvG rfb-50 rph-1*	[25]
MG1655 (Φ24_B_)	MG1655 bearing Φ24_B_ prophage	[26]
MG1655 (Φ24_B_Δ24B_1)	MG1655 bearing Φ24_B_ prophage with deletion of the region encompassing the 80-nt long sequence of the predicted secondary structure of 24B_1	[23]

### Media and growth conditions

For all experiments, *E*. *coli* bacteria were routinely cultured in the Luria-Bertani (LB) medium (EPRO, Władysławowo, Poland) at 37°C with aeration achieved by shaking. The LB broth, supplemented with 0.7% or 1.5% bacteriological agar (BTL, Łódź, Poland), was used as a top or bottom agar, respectively. The Petri dishes with the solid medium were incubated at 37°C for 20 h.

### Preparation of phage lysate

To obtain purified Φ24_B_ or Φ24_B_Δ24B_1 particles, bacteria lysogenic for tested bacteriophages were grown in LB medium to an OD_600_ of 0.1. Then, mitomycin C (EPRO, Władysławowo, Poland) was added to the flask to a final concentration of 1 μg/ml to provoke prophage induction. The mixture was incubated at 37°C on a rotary shaker at the rate of 200 RPM for about 18 h. Following the lysis of host cells, the lysate was treated with 4% chloroform for 15 minutes and cleared of bacterial debris by centrifugation (2000 x *g*, 10 min, 4°C). The supernatant containing phage virions was concentrated in the presence of 10% polyethylene glycol 8000 (PEG8000; EPRO, Władysławowo, Poland) on a magnetic stirrer at 4°C for about 20 h. The homogenous mixture was centrifuged (8000 x *g*, 20 min, 4°C) and the white phage pellet was resuspended in TM buffer (10 mM Tris-HCl, 10 mM MgSO_4_; pH 7.2). The suspension was extracted three times with chloroform (Chempur, Piekary Śląskie, Poland) after centrifugation (2000 x *g*, 10 min, 4°C). Finally, the upper aqueous layer with the virus particles was collected and the phage titer was determined according to the procedure described in the next section. The phage stock was stored at 4°C.

### Determination of the concentration of phage particles in the solution

Phage lysate titration was performed on the standard Petri dishes (Alchem, Toruń, Poland) according to the double overlay plaque assay described by [27]. The top layer was prepared by mixing 2 ml of a prewarmed soft LB agar (0.7%) with 1 ml of the indicator *E*. *coli* MG1655 strain culture. Following supplementation of the mixture with MgSO_4_ (Chempur, Piekary Śląskie, Poland) and CaCl_2_ (Chempur, Piekary Śląskie, Poland) to the final concentration of 10 mM, it was poured onto solid bottom LB agar (1.5%) with a sublethal concentration of chloramphenicol (2.5 μg/ml; EPRO, Władysławowo, Poland). Supplementation of the bottom LB agar with appropriate antibiotic was effective in increasing the size of plaques formed by Stx phages. To determine the number of virus particles per ml suspension (PFU/ml), serial 10-fold dilution was prepared in TM buffer (10 mM Tris-HCl, 10 mM MgSO_4_; pH 7.2). Then, 2.5 μl of each dilution was spotted onto the surface of the top LB agar. Petri dishes were incubated at 37°C for 20 h and the phage concentration was quantified on the basis of the number of plaques.

### Determination of lysis time of host bacteria

Host bacteria were cultivated in LB medium with 10 mM MgSO_4_ (Chempur, Piekary Śląskie, Poland) and 10 mM CaCl_2_ (Chempur, Piekary Śląskie, Poland) at 37°C to an OD_600_ of 0.2. The phage stock solution was added to the flask with bacterial culture to an m.o.i. of 2. The lysis kinetics of infected bacteria were monitored spectrophotometrically by measuring OD_600_ over a period of 300 min. Additionally, the number of bacterial cells per ml (CFU/ml) was determined. To calculate the number of viable bacterial cells after phage infection, 100 μl samples were harvested at appropriate times, and their serial 10-fold dilutions were prepared in 0.85% NaCl (Chempur, Piekary Śląskie, Poland). Then, 40 μl of each dilution was spread onto the bottom LB agar supplemented with ampicillin to a final concentration of 50 μg/ml (EPRO, Władysławowo, Poland). After overnight incubation at 37°C, the bacterial colonies were counted, and the CFU/ml was determined.

### Survival of host bacteria after phage infection in pro-lytic or pro-lysogenic conditions

The procedure described previously was used, with slight modifications [23]. Briefly, host cells were cultured with aeration at 37°C to an OD_600_ of 0.2. Then, 1 ml of the sample was centrifuged (2000 x *g*, 10 min, 4°C) and the pellet was suspended in *(i)* 1 ml of LB medium enriched with 10 mM MgSO_4_ and CaCl_2_ (pro-lytic conditions) or *(ii)* 1 ml of TCM buffer (pro-lysogenic conditions; 10 mM Tris-HCl, 10 mM MgSO_4_, 10 mM CaCl_2_; pH 7.2). Tested bacteriophages were added to the bacterial suspension to an m.o.i. of 5. In the control variant of the experiment, TM buffer (10 mM Tris-HCl, 10 mM MgSO_4_; pH 7.2) was used instead of phage lysate. The mixture was incubated for 15 min at 37°C, and then serial 10-fold dilutions were prepared in 0.85% NaCl (Chempur, Piekary Śląskie, Poland). Then, 40 μl of each dilution was spread onto the bottom LB agar supplemented with ampicillin to a final concentration of 50 μg/ml. Following overnight incubation at 37°C, the fraction of surviving bacteria was calculated relative to the control variant not infected with tested phages.

### Estimation of efficiency of lysogenization

Lysogens among survivors were identified according to the procedure described by [28], with one minor modification. Briefly, bacterial colonies obtained after phage infection were passaged separately, each in a well of a 96-well plate filled with 200 μl of LB medium. The suspensions were incubated with shaking at 37°C to an OD_600_ of 0.1. For estimation of the number of lysogens among tested bacterial cells, mitomycin C was added to each well to a final concentration of 1 μg/ml (this antibiotic had been demonstrated previously to induce prophages in *E*. *coli bacteria* [23, 26, 29]). The plates were then incubated for an additional 3 h. Afterward, 12 μL of chloroform was added to each well and the plates were centrifuged (2000 × *g*, 10 min, 4°C). Then, 2.5 μl of water phase was spotted onto double-layer LB agar supplemented with 2.5 μg/ml of chloramphenicol. The plates were incubated at 37°C for 20 h. A colony was determined as lysogenic if plaques were formed on a bacterial lawn. The efficiency of lysogenization was calculated as a percent of lysogens among all tested bacterial colonies that survived phage infection.

### Intracellular lytic development of tested phages in host bacteria

One-step growth experiments were performed as described previously by [23]. Host bacteria were cultured in LB medium supplemented with 10 mM MgSO_4_ (Chempur, Piekary Śląskie, Poland) and 10 mM CaCl_2_ (Chempur, Piekary Śląskie, Poland) at 37°C to an OD_600_ of 0.2. Then, 10 ml of bacterial suspension was centrifuged (3000 x *g*, 10 min, 4°C) and the obtained pellet was suspended in 1 ml of fresh LB medium with 3 mM NaN_3_ (Merck, Darmstadt, Germany). The phage lysate was added to the host culture to a multiplicity of infection (m.o.i.) of 0.05. Following 10-min incubation at 37°C, the mixture was diluted 10-fold in LB medium supplemented with 3 mM NaN_3_ and centrifuged (3000 x *g*, 10 min, 4°C). The supernatant containing free, unadsorbed virions was discarded and the bacterial pellet was suspended in 1 ml of LB medium with 3 mM NaN_3._ The procedure of centrifugation was repeated 3 times (3000 x *g*, 10 min, 4°C) to remove free phage particles. In the next step, 25 μl of phage-bacteria mixture was added to 25 ml of LB medium (time 0) and cultivated in an incubator shaker at 37°C. The number of infective centers was estimated from samples taken 5 min, 10 min, and 15 min after phage infection by plating under permissive conditions. Samples taken at the indicated times were cleared with chloroform and following the centrifugation step (2000 x *g*, 5 min, 4°C), the number of phage progeny was determined by using the double overlay plaque assay. Petri dishes were incubated at 37°C for 20 h and then burst size was calculated as a ratio of phage titer to the number of infection centers.

### Adsorption experiments

The rate of phage adsorption process was measured according to the procedure described by [30], with some modifications. Overnight cultures of the host strain were 100-fold diluted in LB medium supplemented with 10 mM MgSO_4_ and 10 mM CaCl_2_ and incubated with aeration at 37°C to an OD_600_ of 0.2. Then, the phage stock solution was added to 6 x 10^8^ bacterial cells to an m.o.i. of 0.1. To determine the initial phage titer, the same volume of phage lysate was mixed with 3 ml of LB with 10 mM MgSO_4_ and 10 mM CaCl_2_ (time 0). At given time points, 100 μl samples were collected and added to 900 μl of TM buffer (10 mM Tris-HCl, 10 mM MgSO_4_; pH 7.2). Following centrifugation (12000 x *g*, 1 min, 4°C), the obtained supernatant with unadsorbed phage particles was passed through the filter with a 0.22-μm-pore size SFCA membrane (Thermo Fisher Scientific, Waltham, Massachusetts, USA). Ten-fold dilutions of the supernatant were prepared in TM buffer (10 mM Tris-HCl, 10 mM MgSO_4_; pH 7.2) and full-plate titration was used to calculate the percentage of phage particles adsorbed on the host cell surface. For this purpose, a volume of 25 μl of each dilution was mixed with 1 ml of the overnight culture of the *E*. *coli* MG1655 strain and 2 ml of the top LB agar supplemented with 10 mM MgSO_4_ and 10 mM CaCl_2_. The mixture was gently vortexed for 10 s and poured onto the LB bottom agar containing the sublethal concentration of chloramphenicol (2.5 μg/ml; EPRO, Władysławowo, Poland). The percentage of the adsorbed phages was calculated as follows: ((initial titer—residual titer)/initial titer) x 100.

### Bacterial RNA extraction

For the isolation of total RNA from host cells after infection with tested phage, the previously described procedure was employed [23]. Briefly, *E*. *coli* MG1655 bearing plasmid pUC18 or pUC18_24B_1 was grown in LB medium with shaking at 37°C to an OD_600_ of 0.3. Then, 120 ml of the sample was centrifuged (2000 x g, 10 min, 4°C) and the obtained bacterial pellet was washed once with 30 ml of 0.85% NaCl (Chempur, Piekary Śląskie, Poland). Following centrifugation (2000 x g, 10 min, 4°C), the sample was suspended in 36 ml of the LB medium supplemented with 10 mM MgSO_4_ and 10 mM CaCl_2_. Phage particles were added to the bacterial culture to an m.o.i. of 1.5 and the mixture was incubated with aeration at 37°C. Samples were taken at times 0, 2, 6, and 8 min and treated with Killing buffer (200 mM NaN_3_, 50 mM MgCl_2_, 200 mM Tris-HCl pH 8.0) to inhibit the growth of host bacteria. The total RNA was extracted using the High Pure RNA Isolation Kit (Roche Diagnostics International, Rotkreuz, Switzerland) according to the manufacturer’s protocol. To complete the digestion of genomic DNA from RNA samples, TURBO DNA-free^TM^ Kit (Thermo Fischer Scientific, Waltham, Massachusetts, USA) was employed. The RNA concentration and quality were assessed by a NanoDrop spectrophotometer (Eppendorf, Hamburg, Germany) and agarose gel electrophoresis. Additionally, the absence of DNA was verified by PCR amplification and quantitative real-time reverse transcription-PCR (RT-qPCR) for all tested genes.

### Reverse transcription

cDNA synthesis *via* reverse transcription was performed with 1.25 μg of RNA template, the Transcriptor Reverse Transcriptase, and random hexamer primers (Roche Diagnostics International, Rotkreuz, Switzerland), following the manual supplied by the provider.

### Real-time PCR assay and data analysis

Quantification of transcripts in all tested samples was performed using LightCycler^®^ 480 SYBR Green I Master (Roche Diagnostics International, Rotkreuz, Switzerland) according to the procedure described previously by [23]. The qPCR reaction contained 10 μl 2x SYBR Green I Master, 6.25 ng/μl cDNA, and 200 nM specific primers (Table 2). Real-time PCR amplification was performed with the following amplification program: 95°C for 5 min, followed by 55 cycles of 95°C for 10 s, 60°C for 15 s, and 72°C for 15 s. The *icdA* was selected as a housekeeping gene that showed no changes in expression levels in the presence of tested bacteriophages [31]. No-template controls and a melting curve analysis were performed for each reaction to ensure the specificity of the product. The relative changes in gene expression were analyzed with LinRegPCR using the E-Method with efficiency correction [23, 29].

**Table 2:** Oligonucleotides used in RT-qPCR assay.

Primer name	Sequence (5’→ 3’)
pF_Φ24B_xis	TATCGCGCCGGATGAGTAAG
pR_ Φ24B_xis	CGCACAGCTTTGTATAATTTGCG
pF_Φ24B_cIII	ATTCTTTGGGACTCCTGGCTG
pR_ Φ24B_cIII	GTAAATTACGTGACGGATGGAAAC
pF_Φ24B_N	AGGCGTTTCGTGAGTACCTT
pR_ Φ24B_N	TTACACCGCCCTACTCTAAGC
pF_Φ24B_cI	TGCTGTCTCCTTTCACACGA
pR_ Φ24B_cI	GCGATGGGTGGCTCAAAATT
pF_Φ24B_cro	CGAAGGCTTGTGGAGTTAGC
pR_ Φ24B_cro	GTCTTAGGGAGGAAGCCGTT
pF_Φ24B_cII	TGATCGCGCAGAAACTGATTTAC
pR_ Φ24B_cII	GACAGCCAATCATCTTTGCCA
pF_Φ24B_O	AAGCGAGTTTGCCACGAT
pR_ Φ24B_O	GAACCCGAACTGCTTACCG
pF_Φ24B_Q	GGGAGTGAGGCTTGAGATGG
pR_ Φ24B_Q	TACAGAGGTTCTCCCTCCCG
pF_Φ24B_cat	TCACCAGCTCACCGTCTTTC
pR_ Φ24B_cat	TTCTTGCCCGCCTGATGAAT
pF_Φ24B_S	CTGGGGAGTCTGCTGTTTGG
pR_ Φ24B_S	GCCTTACGCCGGTCTTCTTT
pF_Φ24B_R	GGGTGGATGGTAAGCCTGT
pR_ Φ24B_R	TAACCCGGTCGCATTTTTC
pF_Φ24B_d_ant	TGTTTGCTACCGGGCTGAAT
pR_ Φ24B_d_ant	CCCTTTGCCTGTATCAGCCA
pF_Φ24B_Rz	AACCGTGTTCTGTGTGTGGT
pR_ Φ24B_Rz	GGTTTGTTGCCAGCCACAG
pF_Φ24B_Rz1	CGGATCAACGCCACCTGC
pR_ Φ24B_Rz1	GTTGCATTATCCACGCCGG
pF_Φ24B_icdA	CGAAGCGGCTGACCTTAATTG
pR_ Φ24B_icdA	GTTACGGTTTTCGCGTTGAT

### Metabolite extraction procedure

The extraction of intracellular metabolites from bacterial cells was performed according to the procedure described by [32], with some modifications. Briefly, an overnight culture of bacterial cells was diluted 1:1000 in the fresh LB medium and cultivated at 37°C with shaking. At appropriate time points during growth, 16 units of OD_600_ were harvested and centrifuged (7200 x *g*, 15 min, 4°C). The supernatant was removed, and the obtained pellet was subsequently washed three times with pre-chilled PBS (7200 x *g*, 15 min, 4°C; Thermo Fisher Scientific Inc., Waltham, MA, USA). Finally, the pellet was deeply frozen in liquid nitrogen and suspended in 1 ml of ultrapure water (Thermo Fisher Scientific Inc., Waltham, MA, USA). Bacterial cells were then disrupted by ultrasonication for 1 min at 20% power in an Omni-Ruptor 4000 (OMNI International, Kennesaw, USA) and centrifuged (13000 x *g*, 15 min, 4°C). The obtained cell lysate was filtered through Vivaspin centrifugal concentrator 3KDa (Merck, Darmstadt, Germany) and 448 ul of filtrate was mixed with 50 ul of DSS standard solution (Merck, Darmstadt, Germany) and 2ul of D2O (Merck, Darmstadt, Germany). Each sample prepared in this way was transferred to a separate NMR tube for analysis.

### NMR spectroscopy and quantification of metabolites

A series of 1D NMR spectra were acquired at 298 K on the 700 MHz Bruker Avance-III NMR spectrometer using the stock pulse sequence for a NOESY experiment with presaturation (*noesypr1d*). The combined acquisition delay and acquisition time was set to 5 s with a mixing time of 0.1 s. The final spectrum represented a total of 128 scans. Spectra were processed with minimal solvent suppression and 1.0 Hz exponential line broadening using NMRPipe [33]. Chenomx NMR Suite v9.02 (Chenomx) was used for all subsequent spectral analyses. The metabolites chosen for the survey were selected from the Chenomx 700 MHz database. To be included in the survey, a metabolite had to have at least one peak with an unambiguous chemical shift that could be accurately measured against a DSS standard with a precision of 0.005 ppm against the reference value. Metabolite plots were made with Pro-Fit v7.0.19 (Quantum Software).

### Preparation of RNAs

A 20-nt 24B_1 RNA was chemically synthesized (Metabion, Planegg, Germany; S1 Table). For other RNAs, the DNA templates for the *in vitro* transcription were obtained by primer extension method (Metabion, Planegg, Germany; S2 Table) and transcribed with T7 RNA polymerase as previously described [34]. RNAs were purified by gel electrophoresis and 5′-^32^P labeled using T4 polynucleotide kinase (Thermo Fisher Scientific Inc., Waltham, MA, USA), which was followed by phenol-chloroform extraction, purification on P-30 spin columns (Bio-Rad Laboratories Inc., Hercules, USA), and ethanol precipitation.

### Protein overproduction and purification

The *E*. *coli* Hfq protein was purified as described elsewhere [35].

### Equilibrium Binding Assay

Prior to use, RNAs were denatured by heating at 90°C during 2 min, then refolded on ice for 5 min. The desired range of unlabeled RNA or Hfq concentrations were obtained by serial dilution in the binding buffer (24 mM Tris, pH 7.5, 150 mM NaCl, 5% glycerol, 100 μg/ml BSA, 1 mM MgCl_2,_). Unlabeled RNAs or Hfq protein dilutions were then mixed in a 1:1 ratio with 2 nM ^32^P-labeled RNAs in the binding buffer. The final concentrations were 1 nM of ^32^P-RNA and 100 nM of unlabeled RNA or Hfq at maximum. Incubations were performed for 4 hours for RNA-RNA assays and for 0.5 hour for RNA-Hfq assays at RT in low-protein binding microplates. Afterward, 5 μl aliquots were loaded onto a 6% non-denaturing polyacrylamide gel (19:1) and run in 0.5x TBE buffer. Gels were vacuum-dried, exposed to phosphor screens, and quantified using a phosphorimager (Fujifilm FLA-5000) with ImageQuant software. The equilibrium dissociation constant (*K*_d_) values were calculated by fitting data to the quadratic equation using GraphPad Prism software. Standard deviations were calculated from at least three independent experiments.

### Statistical analysis

The data are expressed as the means ± SD. All experiments were repeated at least three times. Comparisons of two average values were compared by using Student’s *t*-test. Significant differences were marked by asterisks as follows: *p* < 0.05 (*), *p* < 0.01 (**), or *p* < 0.001 (***).

## Results

### Effects of the 24B_1 RNA overproduction on the bacteriophage development in *E*. *coli* bacteria

To test if the overproduction of the small, microRNA-size, 24B_1 molecule can influence the development of bacteriophage Φ24_B_, we constructed an *E*. *coli* bacterial mutant bearing an additional copy of the gene coding for the 24B_1 molecule in the plasmid pUC18. After infection with wild-type phage Φ24_B_, the production of the 24B_1 molecule occurred due to the activity of genes located in both the phage genome and plasmid pUC18_24B_1. Importantly, because of our observation that IPTG, the synthetic inducer of the *lac*-operon in pUC18, used at concentrations even lower than standard 1 mM, negatively affected the growth of *E*. *coli* bacteria used as hosts (S1 Fig), we decided to express the *24B_1* gene from its natural promoter *p*_24B_1_.

**Fig 1:**
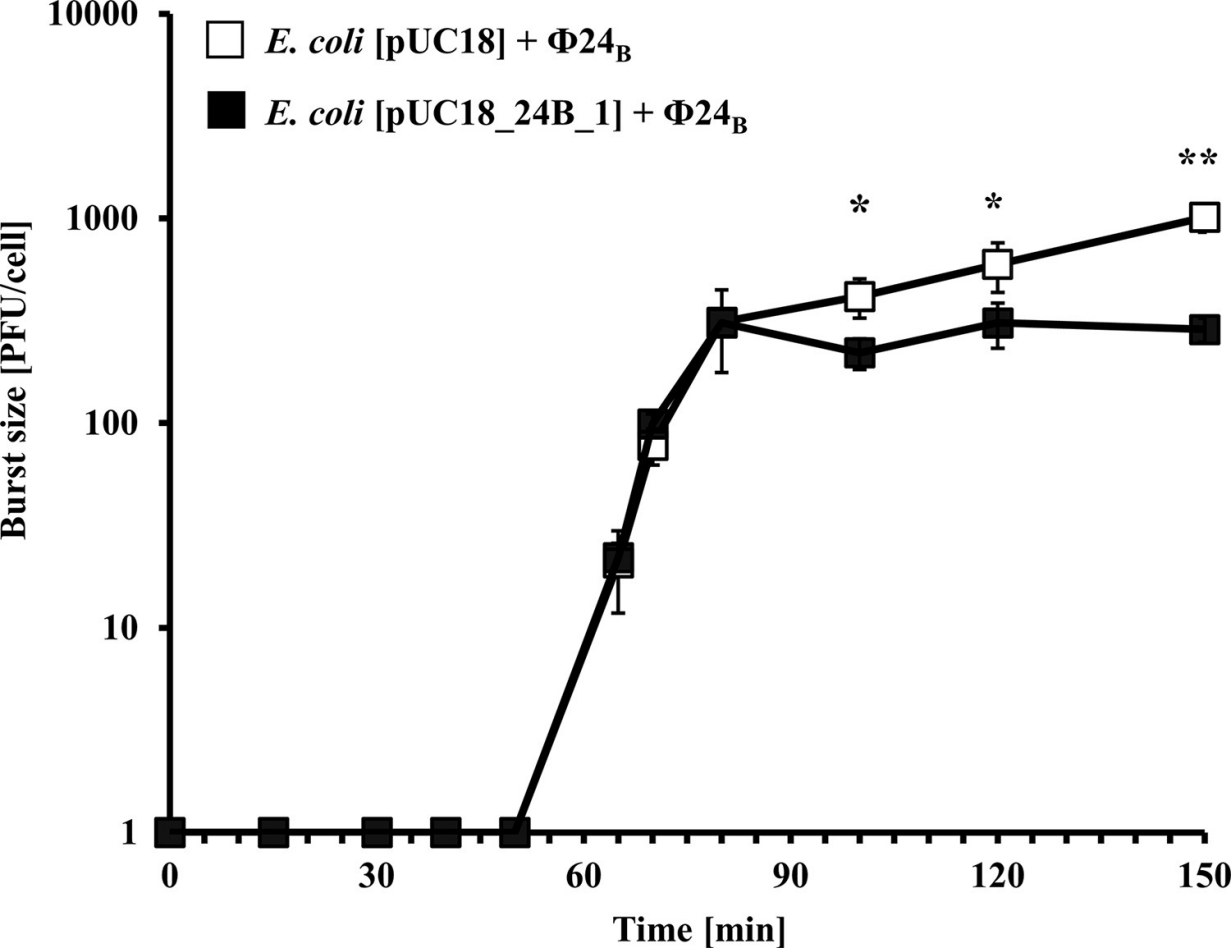
Kinetics of phage progeny production during the intracellular life cycle of Stx phage Φ24_B_ in the *E*. *coli* MG1655 host bearing pUC18 (□) or pUC18_24B_1 (■) plasmids. The presented results are mean values from three biological experiments with error bars indicating SD. Note that in some cases, the error bars are smaller than the sizes of symbols. Statistical analysis was performed by using a Student’s *t*-test. The significance of differences between compared experimental variants is marked by asterisks: *p* < 0.05 (*) or *p* < 0.01 (**).

To assess whether the specific aspects of phage development are targeted by the 24B_1 molecule under its overexpression conditions, we conducted a series of physiological experiments that provided results opposite to those obtained in the case of the phage deletion mutant Φ24_B_Δ24B_1. This mutant was unable to produce the studied sRNA molecule as published by us previously [23]. The measurement of the efficiency of phage progeny production during the lytic cycle occurring after infection of non-lysogenic *E*. *coli* with the phage indicated that this process was less efficient in the bacterial strain bearing an additional copy of the *24B_1* gene on a plasmid relative to the control strain carrying the pUC18 vector (Fig 1).

**Fig 2:**
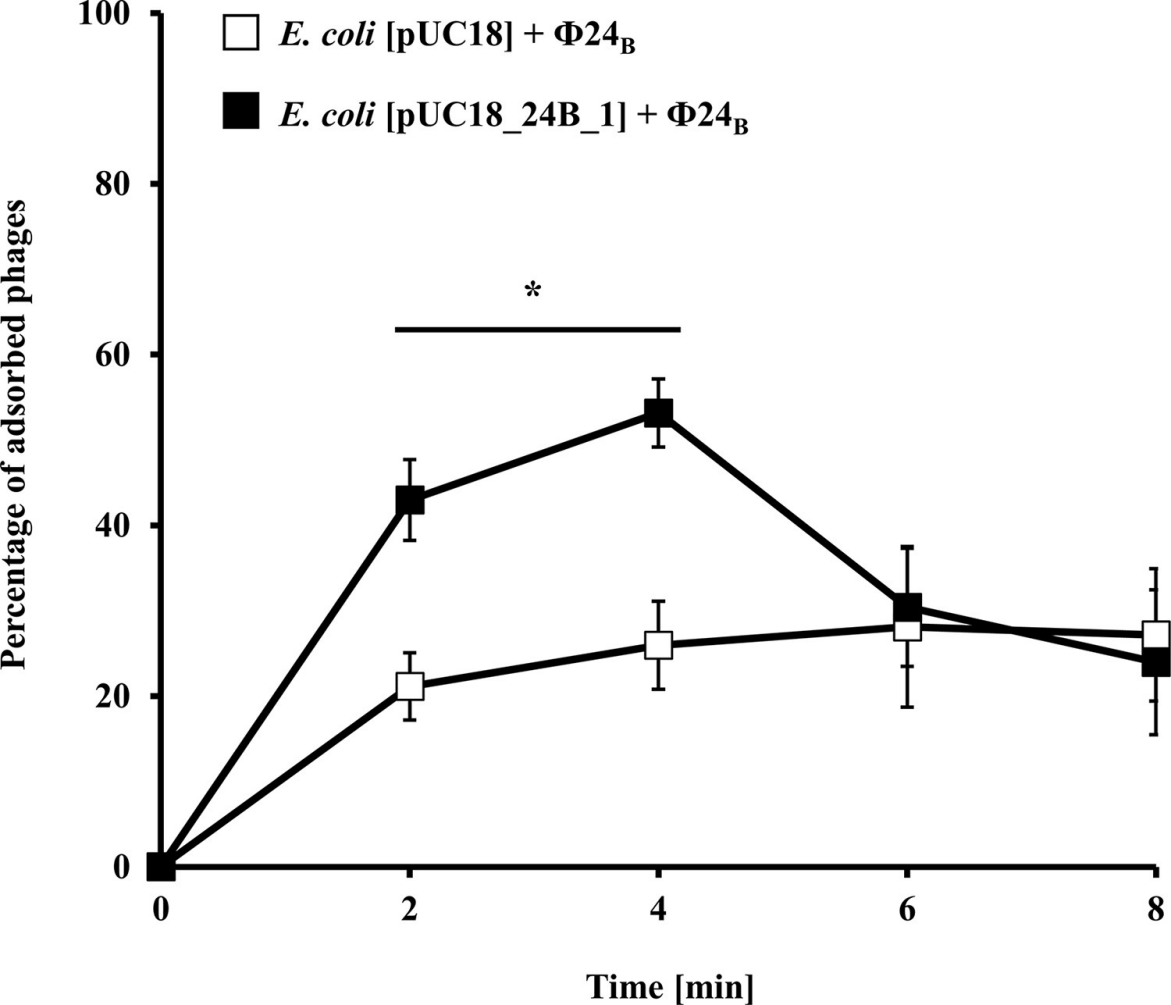
Kinetics of adsorption of Φ24_B_ phage on the surface of *E*. *coli* MG1655 bacterial cells bearing pUC18 (□) or pUC18_24B_1 (■) plasmids. The tested bacteriophage was added to the host suspensions to an m.o.i. of 0.1. The presented results are mean values from four biological experiments with error bars indicating SD. Statistical analysis was performed by using a Student’s *t*-test. The significance of differences between compared experimental variants is marked by asterisks: *p* < 0.05 (*).

Curiously, the adsorption efficiency of the Φ24_B_ phage particles on the host cells MG1655[pUC18_24B_1] bearing additional copies of the *24B_1* gene was higher relative to the control strain MG1655[pUC18] (Fig 2). This difference was significant at the initial minutes of the experiment before the phage entered subsequent phases of development in the bacterial cell.

**Fig 3:**
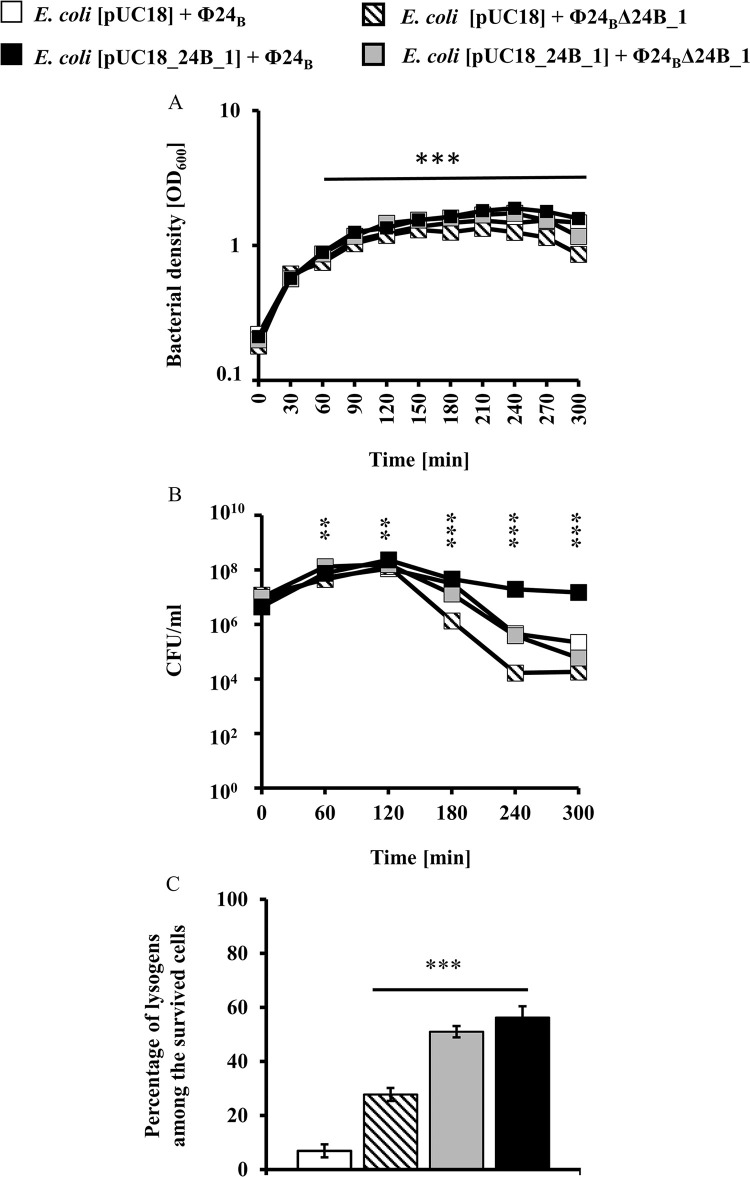
The lysis profile of host bacteria (a), their survivability presented as CFU/ml (b), and efficiency of creation of lysogens at 180 min. (c) after infection of *E*. *coli* MG1655 strain with Stx phages: Φ24_B_ (squares □ and ■) and Φ24_B_Δ24B_1 (triangles ■ and ■) at an m.o.i. of 1.5. Bacterial cells contained either the pUC18 (symbols □ and ■) or pUC18_24B_1 (symbols ■ and ■) plasmids. The presented results are mean values from three biological experiments with error bars indicating SD. Note that in some cases, the error bars are smaller than the sizes of symbols. Statistical analysis was performed by using a Student’s *t*-test. The significance of differences between *E*. *coli* MG1655[pUC18_24B_1] and *E*. *coli* MG1655[pUC18] infected with Φ24_B_ or Φ24_B_Δ24B_1, respectively, is marked by asterisks: *p* < 0.01 (**) or *p* < 0.001 (***).

When comparing the lysis profile of bacterial cultures MG1655[pUC18] and MG1655[pUC18_24B_1] infected with Φ24_B_ or Φ24_B_Δ24B_1 at an m.o.i. = 1.5, we observed that the lysis efficiency was the lowest in the case of the bacterial variant with 24B_1 overproduction. In comparison with the culture lacking the *24B_1* gene (cells bearing pUC18 and infected with the phage deletion mutant Φ24_B_Δ24B_1) the variant with 24B_1 overproduction showed significantly higher optical density OD_600_ values (Fig 3A) and the numbers of survived cells (CFU/ml) (Fig 3B). Interestingly, a similar tendency between these two bacterial variants was revealed in the number of lysogens at 180 min after phage infection. Among cells that survived the infection, we noted 28% and 56% of lysogens of MG1655[pUC18](Φ24_B_Δ24B_1) and MG1655[pUC18_24B_1](Φ24_B_), respectively (Fig 3C).

**Fig 4:**
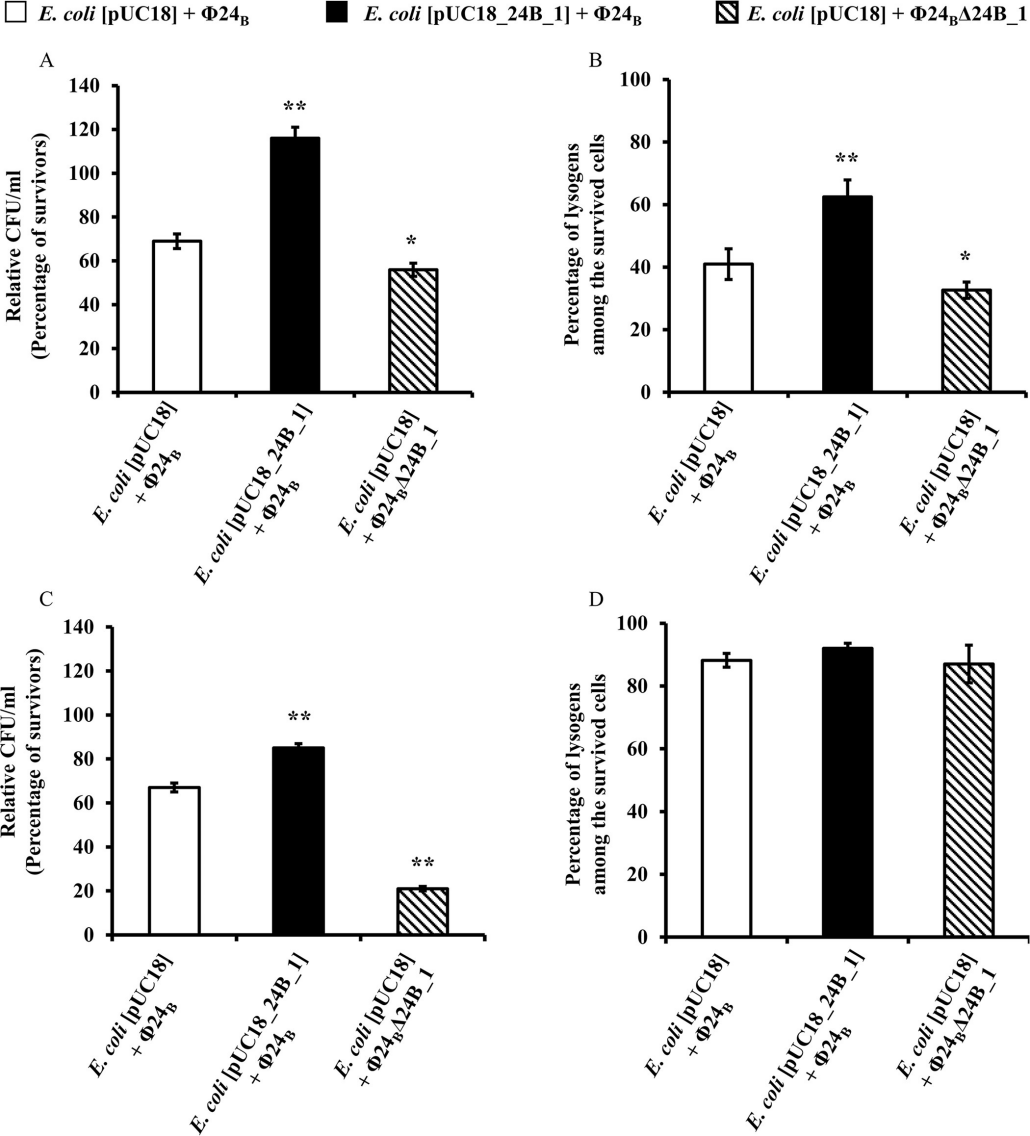
Survival of host bacteria (a, c) and efficiency of creation of lysogens (b, d) after infection of *E*. *coli* MG1655 strain bearing pUC18 or pUC18_24B_1 plasmids with Stx phages: Φ24_B_ and Φ24_B_Δ24B_1 in pro-lysogenic (a, b) or pro-lytic (c, d) conditions. Tested bacteriophages were added to the host suspension to an m.o.i. of 5. Results are presented as mean values ± SD from three independent experiments. Statistical analysis was performed by using a Student’s *t*-test. The significance of differences between compared experimental variants MG1655[pUC18_24B_1] + Φ24_B_ or MG1655[pUC18] + Φ24_B_Δ24B *versus* MG1655[pUC18] + Φ24_B_ is marked by asterisks: *p* < 0.05 (*) or *p* < 0.01 (**).

The significant differences in the number of survived host bacteria MG1655[pUC18] and MG1655[pUC18_24B_1] as well as the number of lysogens appearing after the infection with phage Φ24_B_Δ24B_1 and Φ24_B_, respectively, were also observed at an m.o.i. = 5. These experiments were conducted under pro-lysogenic (poor in nutrients) and pro-lytic (rich in nutrients) conditions. The results obtained under pro-lysogenic conditions (Fig 4A and 4B) indicated that the number of survived bacterial cells (CFU/ml), and the number of lysogens among survivors were significantly higher in strains experiencing 24B_1 overproduction. The controls included MG1655 bacteria bearing vector pUC18 either *(i)* infected with wild-type phage Φ24_B_, thus presenting the natural, unchanged level of 24B_1 expression, or *(ii)* infected with the phage deletion mutant Φ24_B_Δ24B_1, thus having the 24B_1 production turned off completely. These results together demonstrate that lysogenization is favored by additional copies of the *24B_1* gene. Under pro-lytic conditions, the fraction of bacterial cells (CFU/ml) surviving the infection was also significantly higher when overproduction of 24B_1 occurred. It is worth noting that in this case, prophages were formed at similar frequencies in all tested variants (Fig 4C and 4D). Importantly, in relation to the control variants of cells non-treated with phages, the number of cells that survived the infection in all tested variants (MG1655[pUC18] + Φ24_B_; MG1655[pUC18_24B_1] + Φ24_B_; and MG1655[pUC18] + Φ24_B_Δ24B_1) was lower under rich, pro-lytic conditions (approximately 65, 80 and 20% of the not treated with phages controls, respectively) than under poor conditions (approximately 70, 115 and 50% of the controls, respectively). These observations suggest that under pro-lytic conditions the total number of the survived cells is generally lower than under pro-lysogenic conditions, and the majority of them (average 90%) are lysogens (Fig 4D).

### Bacteriophage gene expression profiles in the presence of additional copies of the *24B_1* gene

**Fig 5:**
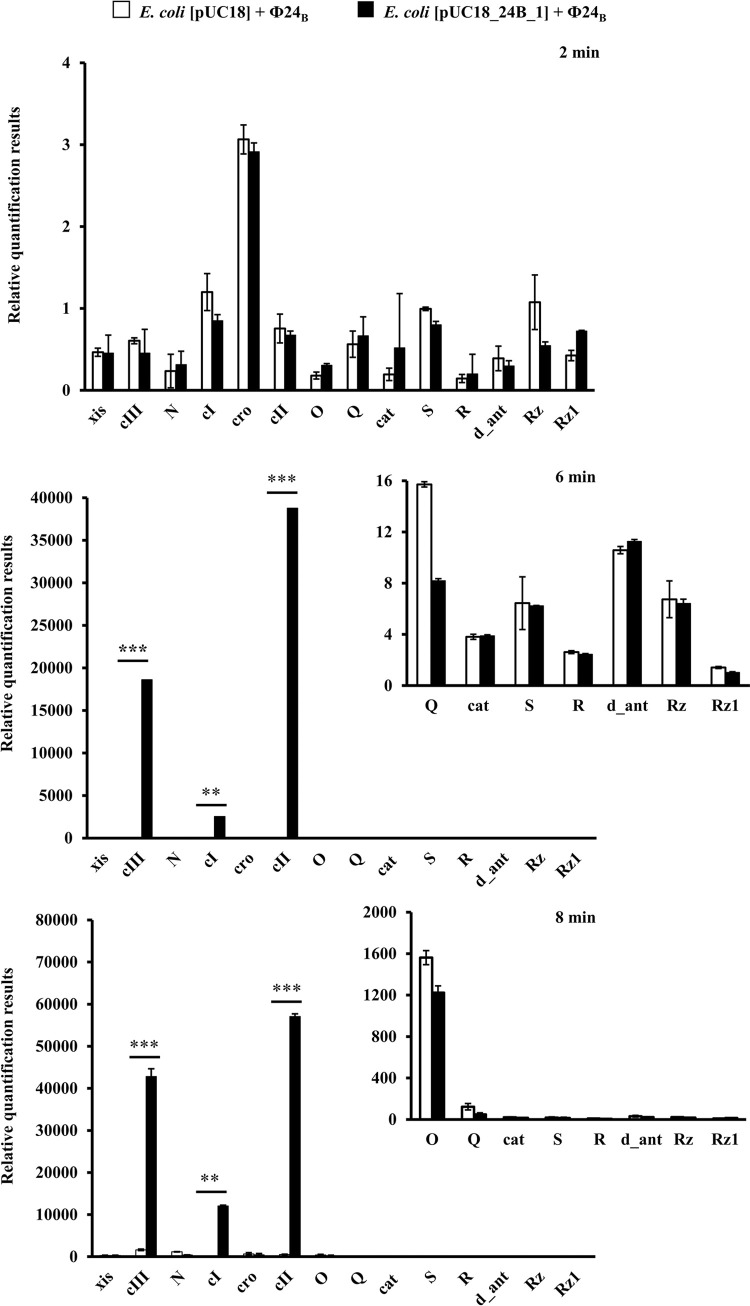
The expression levels of selected bacteriophage genes assessed by RT-qPCR after infection of host bacteria bearing pUC18 (□) or pUC18_24B_1 (■) plasmids with phage Φ24_B_ at an m.o.i. of 1.5. The mRNA levels corresponding to particular genes were determined at the following times after infection: 2, 6, and 8 minutes. The presented results are mean values from three biological experiments with error bars indicating SD. Statistical analysis was performed by using Student’s *t*-test. The significance of differences between compared experimental variants is marked by asterisks: *p* < 0.01 (**) or *p* < 0.001 (***).

A reverse transcription quantitative real-time PCR (RT-qPCR) assay was performed to measure levels of Φ24_B_ transcripts when 24B_1 was overproduced. At 2 min post-infection, some of the selected phage genes were expressed at similar levels in both hosts MG1655[pUC18] and MG1655[pUC18_24B_1]. Contrary to the initial time, drastic differences in levels of a few phage mRNAs were observed at later times. Within the interval of 6–8 min, expression of *c*I, *c*II, and *c*III genes increased substantially when 24B_1 was overproduced relative to a control culture with a natural level of Φ24_B_ synthesis (Fig 5).

It is worth noting that all of the highly expressed genes are associated with the lysogenic pathway. CI is the main repressor of the phage lytic development that inhibits the expression of early phage genes from *p*_R_ and *p*_L_ promoters. CII stimulates the expression of the *c*I gene and the production of an integrase, another protein participating in the lysogenic cycle. Finally, CIII assists with the regulation of CII activity, being an inhibitor of the CII-degrading protease [3].

### The 24B_1 sRNA—mediated metabolic changes in bacteria

**Fig 6:**
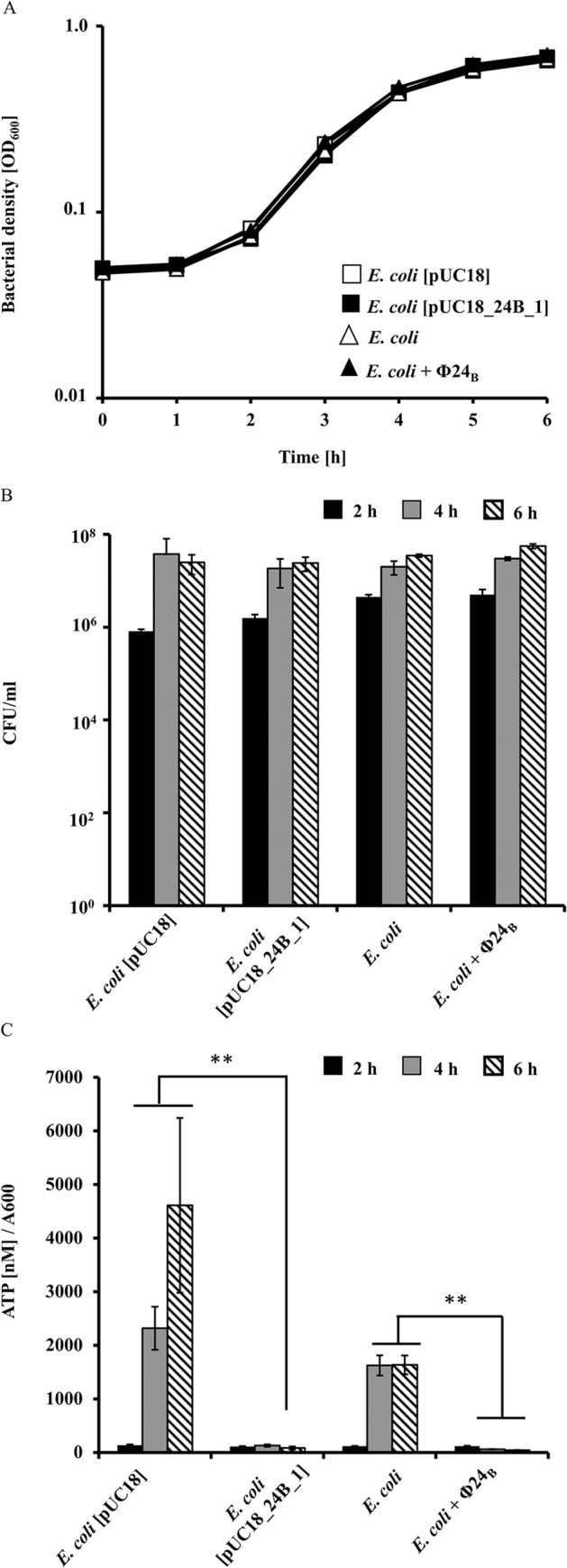
The bacterial growth kinetics (a), number of live bacterial cells presented as CFU/ml (b), and the ATP level (c) measured in *E*. *coli* control strains devoid of the 24B_1 molecule gene (MG1655 and MG1655[pUC18]), and in bacterial strains able to produce 24B_1 molecule form plasmid (MG1655[pUC18_24B_1]) or phage genome (MG1655(Φ24_B_)). Statistical analysis was performed by using a Student’s *t*-test. The significance of differences between *E*. *coli* MG1655[pUC18] and *E*. *coli* MG1655 or *E*. *coli* MG1655[pUC18_24B_1] and *E*. *coli* MG1655 infected with Φ24_B_ are marked by asterisks: *p* < 0.01 (**).

We hypothesized that widespread changes in gene expression associated with 24B_1 would also be manifested by changes in bacterial metabolism. We began this investigation by measuring the effects of 24B_1 on ATP levels. In comparison to variants lacking the 24B_1 sequence (MG1655[pUC18] and MG1655 strains), the 24B_1 molecule produced from its natural promoter *p*_24B_1_ (from both plasmid and phage genome) significantly decreased ATP levels in *E*. *coli* during the 4–6 hour interval of the experiment. Importantly, the bacterial growth rate and the number of live bacterial cells were similar in all tested variants thus excluding the possibility that the observed ATP level depends on the cell number (Fig 6).

**Fig 7:**
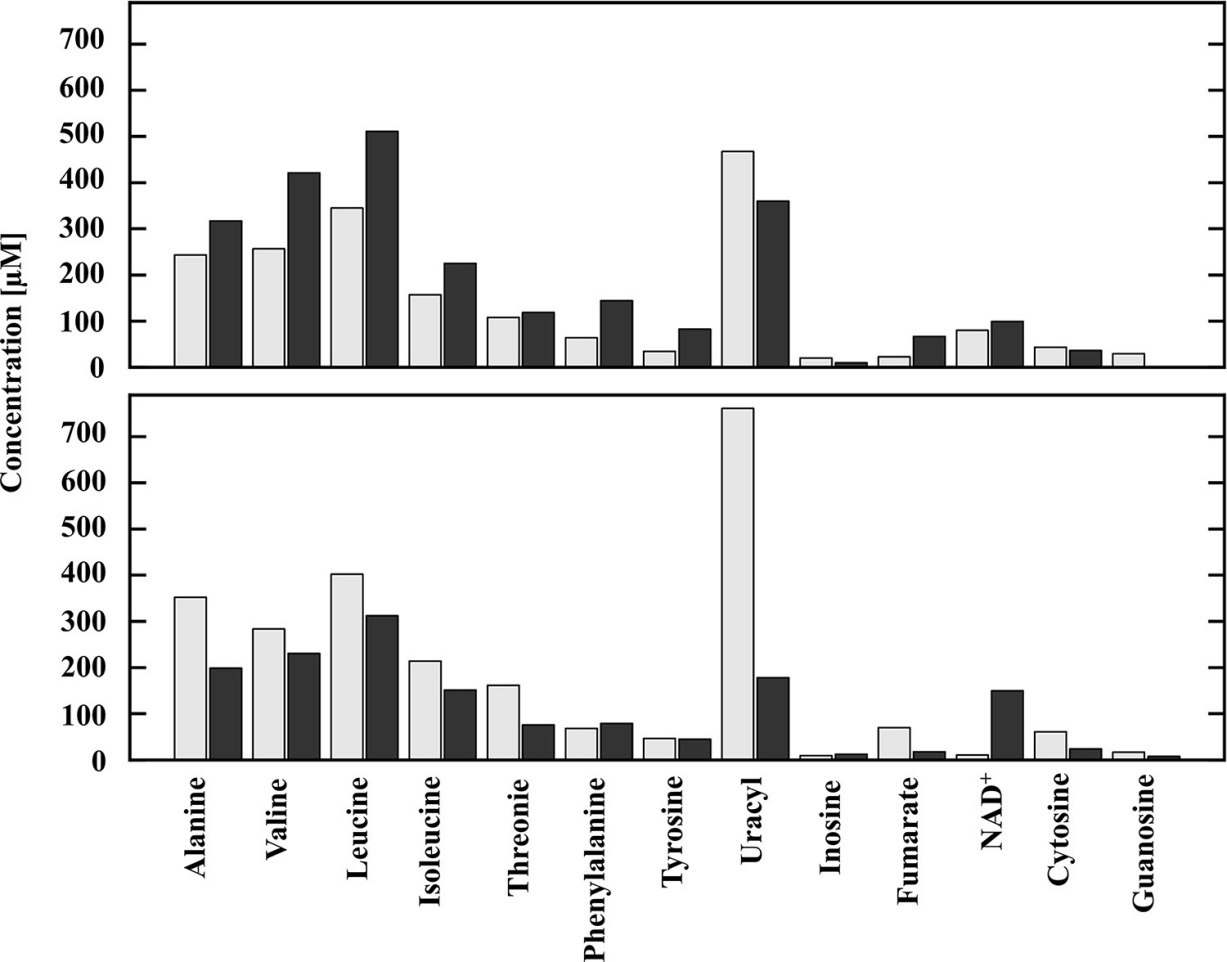
The metabolite profile of *E*. *coli* bacteria carrying pUC18 (light bars) and pUC18_24B_1 (dark bars) at four hours (upper panel) and six hours (lower panel) of the experiment. The results are the average of three repetitions and standard deviations do not exceed 10%.

Building upon the observation that ATP levels were different between variants producing 24B_1 molecule and those lacking its sequence, we measured a set of thirteen metabolites according to their unique chemical shifts in a 1D NMR spectrum (Fig 7). These metabolites could be categorized into aliphatic amino acids (alanine, threonine, valine, leucine, isoleucine), aromatic amino acids (phenylalanine, tyrosine), nucleobases (inosine, uracil, cytosine, guanosine), and energy production (NAD^+^, fumarate). The experiments were designed to sample metabolites at 4 hours and 6 hours of growth from a control MG1655 *E*. *coli* strain carrying an empty vector and an experimental strain with a plasmid supporting 24B_1 production. Unfortunately, a determination of ATP levels could not be accurately performed using the NMR method, because the adenine base chemical shifts at ~8.0 ppm could not be unambiguously assigned and integrated.

Comparing the 4-hour and 6-hour samples for the control plasmid in which no 24B_1 was overproduced, the metabolite levels were more or less stably maintained, especially for the most of amino acids. Over the period sampled, the metabolic profile of cultures overproducing 24B_1 experienced more changes. There was an excess of amino acids in the 4-hour sample that is diminished by the 6-hour sample, perhaps as the 24B_1 RNA continues to accumulate and the culture shifts from the exponential growth phase to the stationary phase.

NAD^+^ and fumarate have unambiguous chemical shifts in all NMR spectra making these compounds useful for diagnostics of energy usage. A representative NMR spectrum is available in the Supporting information as S2 Fig. Within the citric acid cycle, NAD^+^ is an electron carrier. Fumarate is the last substrate in the citric acid cycle to carry a double bond which is converted to malate upon the addition of water. Thus, if the efficiency or output of the citric acid cycle is diminished, there would be less fumarate available and an accumulation of NAD^+^ that is not being converted to NADH throughout the cycle. The reciprocity between fumarate and NAD^+^ appears to be enhanced when 24B_1 is overproduced.

### The *sdhB* transcript as a target for the mature 24B_1 RNA molecule

Following our key observation that 24B_1 produced from its natural promoter *p*_24B_1_ significantly decreased the ATP levels in *E*. *coli*, we hypothesized that the citric acid cycle may be impaired. This hypothesis was further supported by the decrease in the levels of fumarate and increased levels of NAD^+^ as determined by the NMR metabolomics study. Examining the idea that fumarate may be a key metabolite in addition to ATP, we compared the sequences of *sdhCDAB* genes that encode the individual subunits of succinate dehydrogenase Sdh. The sole membrane-bound Sdh enzyme participates in the citric acid cycle converting succinate to fumarate and takes part in the aerobic electron-transport pathway to generate energy *via* oxidative phosphorylation reactions. The bioinformatics analysis of the *sdhCDAB* genes sequences revealed the potential binding site for 24B_1 within the *sdhB* gene coding for the FeS subunit of succinate dehydrogenase. This binding site is located at the 5ʹ end of the *sdhB* coding sequence between nucleotides 7–26 and was predicted by IntaRNA software [36] with a high probability energy score (-10.56 kcal/mol) and hybridization energy value (-15.17 kcal/mol), as shown in S3 Fig. Importantly, only interaction energies below or equal to 0 are considered and such interactions are visualized by the IntaRNA program. A seed region typical for eukaryotic microRNAs was predicted by IntaRNA at positions 7–13 from the 5´-end of 24B_1 molecule. In eukaryotic cells, the seed sequence is essential for the binding of the microRNA to the target mRNA and has to be perfectly complementary.

**Fig 8:**
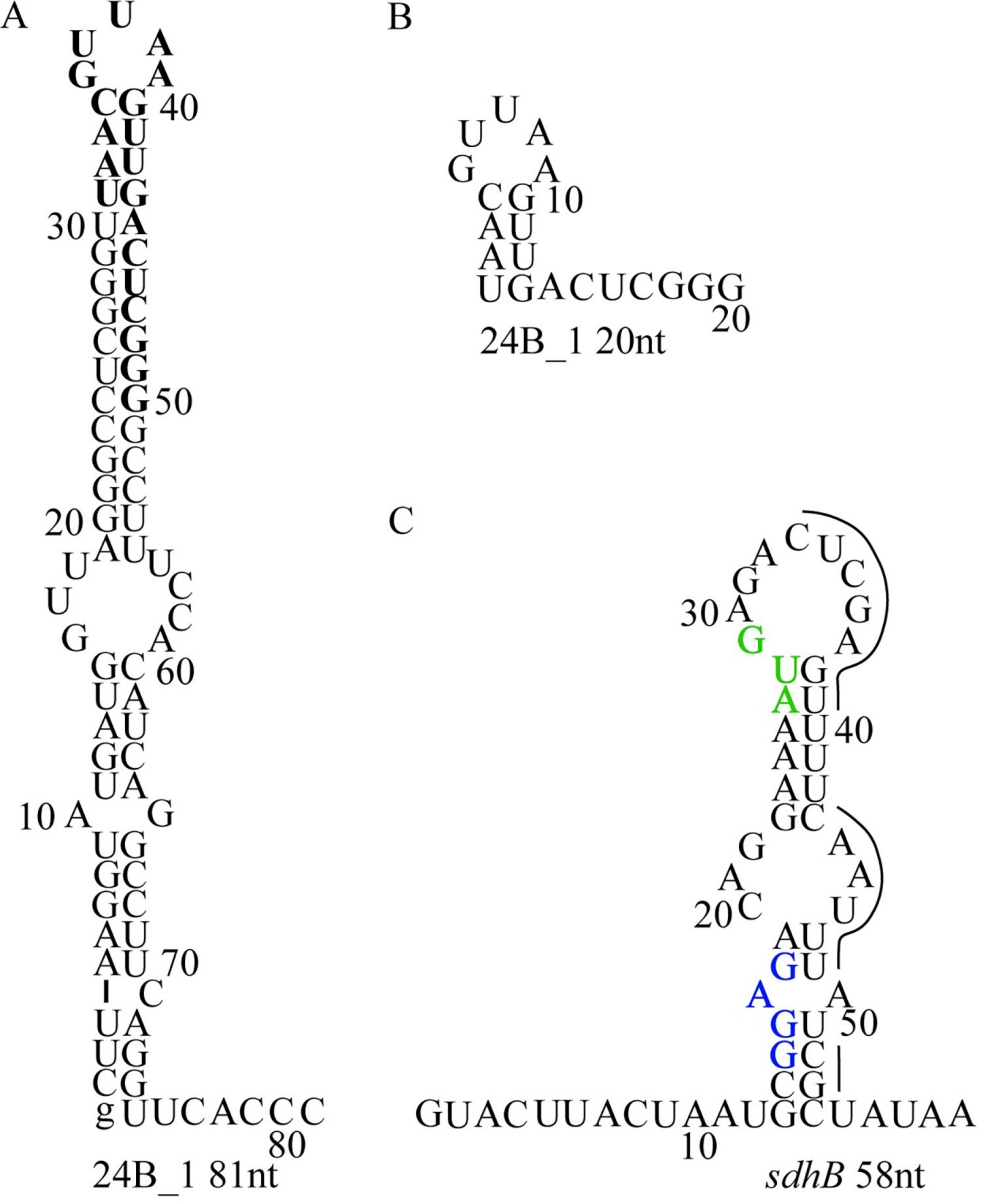
The secondary structures of the 81-nt long precursor of 24B_1 RNA (a), the mature 20-nt long 24B_1 RNA (b), and the sequence of the intergenic region and beginning of the coding sequence of *sdhB* gene (c), which were used in this study. The lower case g denotes guanosine residue added on the 5′ end of 81-nt long 24B_1 RNA precursor to enable T7 RNA polymerase transcription. The sequence of the 20-nt long 24B_1 RNA within its precursor is marked in bold font (a). The Shine-Dalgarno sequence within the 5ʹ-UTR of *sdhB* mRNA is marked in navy blue, the AUG start codon in green, and the sequence of *sdhB* mRNA predicted by IntaRNA as complementary to the mature 24B_1 RNA is marked with a line (c). The secondary structures were predicted using *RNAstructure* software [37].

**Fig 9:**
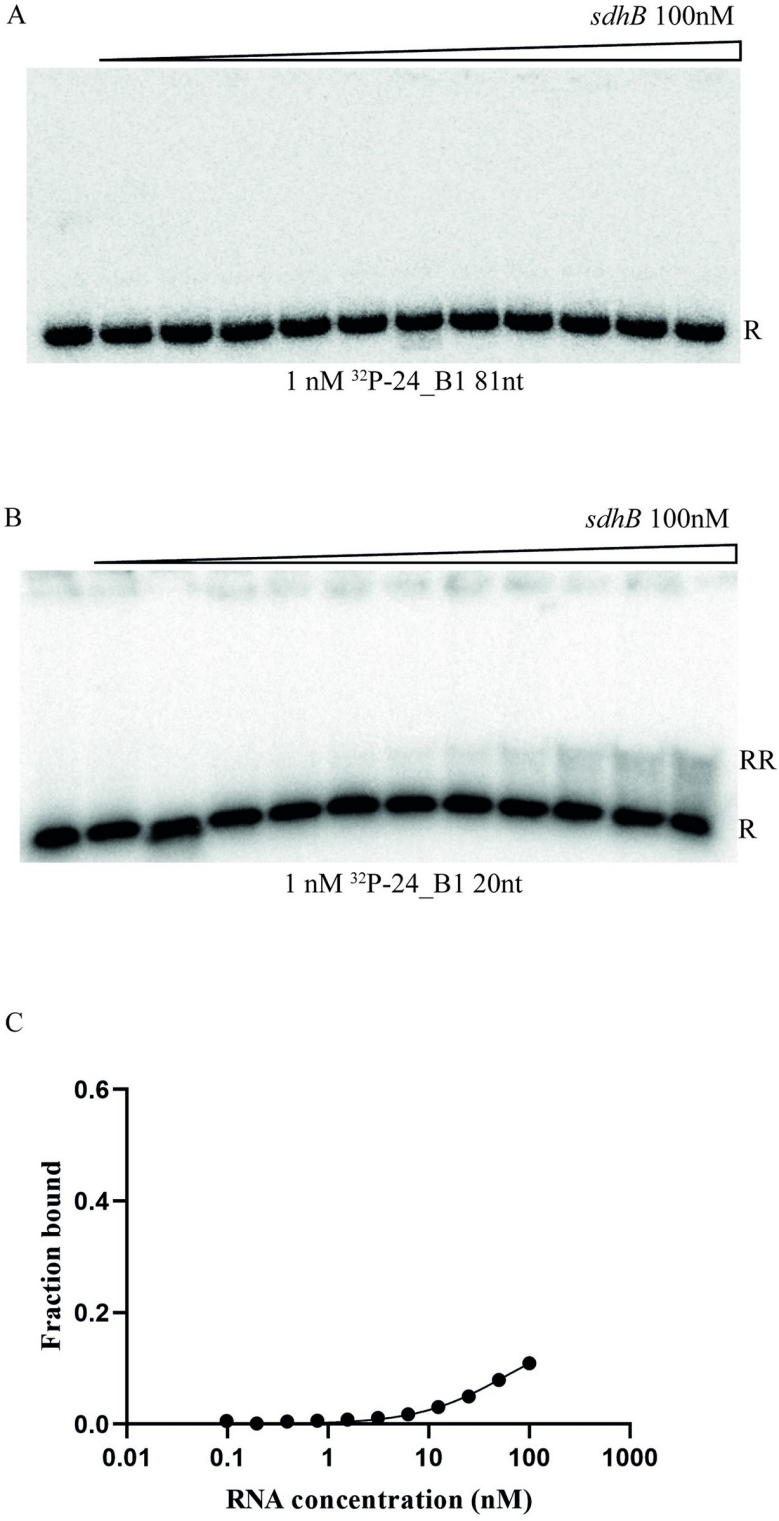
The mature 20-nt long 24B_1 RNA base-pairs with the 5ʹ-UTR of sdhB mRNA. The pairing of the ^32^P-labeled 81-nt long precursor of 24B_1 RNA (a) and mature 20-nt long 24B_1 RNA (b) to the 5ʹ-UTR of *sdhB* mRNA was monitored using a gelshift assay. The fraction bound data from (b) were plotted *versus* RNA concentration in (c). Free ^32^P-RNA is marked as R, and RNA-RNA complex as RR. The average fraction of mature 24B_1 RNA bound at the maximum 100 nM concentration of *sdhB* mRNA, calculated from three independent experiments, was 8 ± 5%.

To test whether mature 24B_1 or its precursor RNA can interact with the predicted pairing site at the 5’ region of the coding sequence of *sdhB* gene we measured the binding of purified RNAs using an *in vitro* gelshift-based assay (Figs 8 and 9). The predicted structure of the 80-nt long precursor 24B_1 RNA showed that the sequence corresponding to the short 24B_1 is located in the apical portion of the long hairpin structure formed by the precursor RNA. This sequence is involved in a stable double-stranded structure, which would limit its availability for pairing to another sequence (Fig 8A). On the other hand, the 20-nt long mature 24B_1 RNA was predicted as less structured, although it still contained a short 4-basepair double helix (Fig 8B). Additionally, in this study we used a 56-nt long mRNA fragment including the untranslated region upstream of the *sdhB* mRNA and the beginning of its coding sequence. This mRNA fragment is predicted to form a secondary structure, in which the sequence complementary to 24B_1 is base-paired with a region including the Shine-Dalgarno sequence and the start codon of *sdhB* mRNA (Fig 8C).

When we compared the binding of the 24B_1 precursor and the mature 24B_1 to the 56-nt long fragment of the *sdhB* mRNA, we found that the mature 24B_1 molecule formed a complex with the *sdhB* mRNA, although the fraction bound at the maximum 100 nM mRNA concentration used was less than 10%, which did not allow calculating the binding affinity (Fig 9B and 9c). On the other hand, the binding of the 24B_1 precursor RNA to the *sdhB* mRNA fragment was not detected (Fig 9A). These data suggest that the mature 24B_1 RNA is more likely to interact with other RNA molecules than the precursor RNA because its structure is less stable and, hence, it is more available for pairing with other RNAs. In particular, these data show that the mature 24B_1 RNA can base-pair with the complementary sequence of the *sdhB* mRNA. The fact that the binding is not strong in the *in vitro* assay may indicate that the conformations of the interacting RNAs are more favorable for binding in the conditions *in vivo* or that other factors affect the RNA structure *in vivo*, thus influencing the binding.

### Hfq binds the 24B_1 precursor form

**Fig 10:**
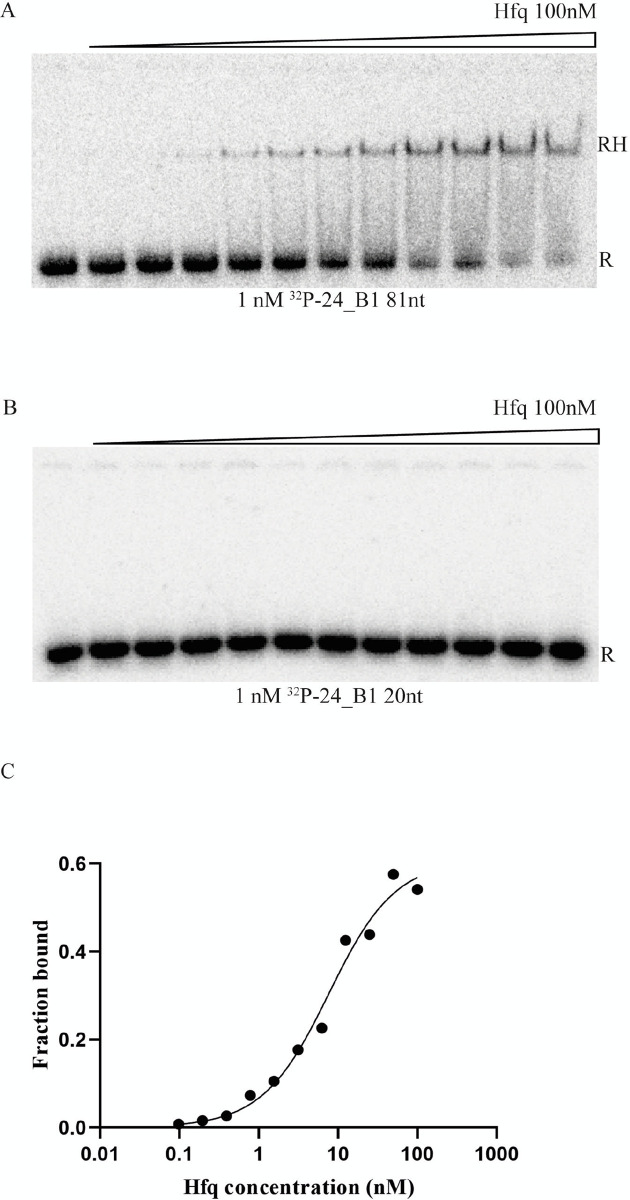
The Hfq protein binds to the 81-nt long precursor of 24B_1 RNA, but not to the mature 20-nt long 24B_1 RNA. The equilibrium binding of ^32^P-labeled 81 nt-long precursor of 24B_1 (a), and the mature 20-nt long 24B_1 RNA (b) to the Hfq protein was monitored using a gelshift assay. Free ^32^P-labeled RNAs are marked as R, and the RNA-Hfq complex as RH. The raw binding data from (a) were fit using the quadratic equation in (c). The average *K*_d_ value of 25 ± 13 nM was calculated from three independent experiments.

To further characterize the properties of the mature 24B_1 RNA and its precursor, we compared their binding to *E*. *coli* Hfq protein, which is known to bind many small bacterial RNAs [38, 39]. Interestingly, the data showed that Hfq did bind to the precursor RNA with a *K*_d_ value of 25 nM. On the other hand, its binding to the mature 24B_1 RNA was not detected (Fig 10). The apical portion of the 24B_1 precursor RNA hairpin is stably base-paired, and, hence, would not be expected to bind this protein. However, the lower part of the structure, below the base-pair G_21_-C_53_, is likely less stable and contains an U-rich internal loop, which could potentially serve as a binding site for Hfq (Fig 8A). Overall, these data suggest that Hfq could affect the stability or processing of the precursor 24B_1 RNA, but not the mature 24B_1 RNA. Hence, it is possible that mature 24B_1 RNA does not depend on RNA-binding proteins for its function, similar to *cis*-encoded antisense RNAs, which are not dependent on Hfq for pairing with their RNA targets [40].

## Discussion

When considering the significance of small regulatory RNA molecules derived from phages, it becomes evident that they play a crucial role in the development of both the phage and its host [41]. In bacteria, these sRNAs have a profound impact on various processes such as virulence, colonization ability, motility, and cell growth or death [40]. Similarly, in the case of phages, they play vital roles during the early stages of infection, maintaining the state of lysogeny and suppressing the expression of late structural genes [42, 43].

The small RNA molecule, 24B_1, investigated in this work and originating from the Stx phage, deserves special attention in this context. While other microRNA-size, phage-encoded regulatory RNAs have not been described yet, similar molecules are well-documented in viruses that infect eukaryotic cells, particularly herpesviruses [44, 45]. Notably, this sRNA shares several similarities with herpesviral microRNAs. Importantly, like Stx phages, herpesviruses undergo two distinct stages in their lifecycle and are able to cause latent or lytic infections and switch between these states. Similar to phage lysogeny, viral latency represents a specialized form of persistent infection where most viral genes remain silenced. Herpesviruses can reactivate and transit to the lytic stage, leading to host cell lysis and the production of infectious viral progeny, which spreads to new cells [46].

Comparable to the identified herpesviral microRNAs, 24B_1 is a very short RNA molecule, only 20 nucleotides in length, produced through the cleavage of a precursor (80-nt long) transcript and likely undergoing a multi-step maturation process. Importantly, only the precursor form of 24B_1 binds the bacterial RNA-binding protein called Hfq (Fig 10). The functional roles of Hfq in sRNA action are fairly well understood and widely described in the literature [38, 39, 47, 48] however, we show for the first time that this protein is able to bind the precursor form of phage 24B_1 molecule. On the other hand, the performed analysis did not confirm the binding of the short (20-nt long) 24B_1 with Hfq protein. This result suggests that similar to microRNAs produced in eukaryotic cells, different proteins may participate at the particular stages of maturation and operation of such small RNA molecules in prokaryotic cells. Furthermore, like most identified herpesviral microRNAs, 24B_1 demonstrates limited complementarity with its identified target—the *sdhB* gene and actively promotes the phage’s lysogenic state, which was confirmed under 24B_1 deletion and overproduction conditions (this work). Importantly, the imperfect pairing of the SdhB transcript to the 24B_1 seed region can be compensated by extended pairing interactions with the 3’ end of the 24B_1 molecule, and this phenomenon is also quite frequently observed in the case microRNAs functioning in eukaryotic cells [49].

In herpesviruses, small regulatory RNAs classified as microRNAs have been identified as key players in the maintenance of viral latency [50]. The examples are miRNAs of the murine gammaherpesvirus-68 (MHV-68) which contribute to the maintenance of latent infection *in vivo*. The investigation of mutated viruses has unveiled that the presence of MHV-68 miRNAs are not necessary for the short-term replication of the virus, but they play a crucial role in establishing a persistent infection within memory B cells, ensuring a lifelong presence [51]. In turn, miRNAs of Kaposi’s sarcoma-associated herpesvirus (KSHV), such as miR-K12-11 and miR-K12-3, prevent lytic reactivation by reducing the expression of cellular transcription factors MYB, C/EBPα, and Ets-1 [52, 53]. The prevailing model suggests that during latency, viral microRNAs are expressed at relatively high levels, facilitating this phase of infection and thus negatively impacting the expression of lytic genes. Otherwise, during the lytic cycle, the transcripts liberate from inhibition by these microRNAs and changes occur in their transcriptional activity. As a result, although most virus-encoded microRNAs are present and detectable during the lytic phase, their role becomes diminished during this stage of infection [54]. Interestingly, as described by us previously [23], the 24B_1 sRNA has been identified during phage lytic development, 80 minutes after prophage induction with mitomycin C. What is more, similar to viral microRNAs, the 24B_1 sRNA plays a significant role in maintaining the phage in the form of prophage. This is supported by previous results obtained for *24B_1* phage deletion mutant [23], and also under conditions of 24B_1 overexpression (this study). Here, we demonstrate that an excess of the 24B_1 molecule in the cell leads to a decrease in the efficiency of bacterial culture lysis after infection with Stx phage, and thus an increase in both the number of cells that survived the infection with phage, as well as the number of lysogens among survivors. The physiological effects were accompanied by increased levels of *c*I, *c*II, and *c*III mRNAs which protein products are strongly associated with the lysogenic pathway. Importantly, this effect seems to be indirect as we did not find significant binding sites for the 24B_1 molecule within these genes. In this regard, the 24B_1 small RNA closely resembles eukaryotic microRNAs of viral origin.

In the context of persistent viral infections, the role of miRNAs encoded by herpesviruses is of utmost importance, influencing various aspects such as the metabolic processes in host cells [53]. Notably, miRNAs of Epstein-Barr virus (EBV) and KSHV disrupt the balance of metabolic processes through direct targeting of key proteins or indirect regulation of multiple signalling pathways. Recent research has shed light on the impact of several EBV miRNAs on metabolic processes of cancer cells. For instance, the expression of miR-BART1 significantly upregulates the levels of phosphoglycerate dehydrogenase (PHGDH) in an NPC cell line called CNE1 [55]. PHGDH is a critical enzyme involved in the synthesis of serine and glycine, central metabolites that play essential roles in various biosynthetic pathways [56]. Regarding KSHV, it has been observed that viral miRNAs affect host cell energy metabolism through the regulation of multiple pathways. Firstly, KSHV miRNAs stabilize and activate the transcription factor HIF1α-a master regulator of cell metabolism, by targeting EGLN2, a hypoxia-inducible factor prolyl hydroxylase. Secondly, KSHV miRNAs downregulate the mitochondrial heat shock protein A9 (HSPA9), which leads to a decrease in mitochondrial copy numbers and promotes anaerobic glycolysis (commonly known as the Warburg effect). The downregulation of EGLN2 and HSPA9 enables cancer cell proliferation even under low oxygen conditions [57]. In the field of oncology, the Warburg effect refers to the phenomenon observed in many cancer cells, where energy production primarily relies on anaerobic glycolysis rather than the typical citric acid cycle and oxidative phosphorylation occurring in the mitochondria of normal cells. This process involves high glucose uptake, followed by glycolysis and lactic acid fermentation in the cytosol, even in the presence of ample oxygen. While fermentation produces ATP with lower efficiency compared to the citric acid cycle and oxidative phosphorylation, it allows proliferating cells to utilize nutrients like glucose and glutamine more effectively for biomass production. Besides, it allows for bypassing the catabolic oxidation of nutrients into CO_2_ and thus promotes anabolic processes [58, 59]. Surprisingly, the overproduced 24B_1 molecule seems to induce a similar effect in prokaryotic cells. As we have shown in this work, its overproduction caused a decrease in the level of ATP and fumarate, but stimulated an increase of NAD^+^, which is most likely not converted to NADH. This is most likely due to the negative regulation of the succinate dehydrogenase subunit gene (*sdhB*) by 24B_1 as *in vitro* binding of these two RNA transcripts has been observed. Sdh is the enzyme that participates in the citric acid cycle converting succinate to fumarate and takes part in the aerobic electron-transport pathway to generate energy *via* oxidative phosphorylation, thus is essential for the aerobic utilization of carbon sources. As reported recently, the Arg244His mutation in the amino acid sequence of the SDHB subunit attenuated ATP production and reduced mitochondrial number in *Caenorhabditis elegans*. These Arg244His SDHB mutants demonstrated elevated lactate/pyruvate levels, pointing to a missense-induced, Warburg-like aberrant glycolysis. Authors concluded that mutation in the amino acid sequence of SDHB rewires metabolism, resembling metabolic reprogramming in cancer [60]. Similar observations have been reported by other scientists who showed strong evidence that some mutations in SDH impair oxidative phosphorylation, which highly contributes to the Warburg metabolism switch observed in cancer cells [61–63]. The advantage of the Warburg phenomenon in cancer cells has been unclear however it was proposed that the metabolism of cancer cells, and indeed all proliferating cells, is adapted to facilitate the uptake and incorporation of nutrients into the biomass (e.g., nucleotides, amino acids, and lipids) needed to produce a new progeny of cells. Thanks to this, cancer cells metabolize nutrients in a manner conducive to proliferation rather than efficient ATP production [58]. Does the presence of the prophage in a bacterial cell cause a similar effect? Recent results obtained by Holt and collaborators [64] showed that integration of phage Φ24_B_ into bacterial genome increases cell proliferation. When grown under normal conditions, both the single and double *E*. *coli* lysogens exhibited a considerable increase in cell numbers (exceeding 200%) compared to the MC1061 strain without Φ24_B_ prophage [64]. Phage-mediated metabolic reprogramming of the bacterial cell was also presented by [65]. The authors showed that infection of the marine bacterium *Pseudoalteromonas* with PSA-HP1 phage alters central carbon and energy metabolisms. Their findings indicate that the glyoxylate shunt was used in phage-infected cells instead of the classical citric acid cycle. Importantly, all *sdh* genes (*sdhABCD*) were downregulated 40 and 60 minutes after phage infection [65].

Undoubtedly energetic status is a significant modulator of lysogeny. The presence of a low ATP concentration within the intracellular environment creates a favorable condition for the accumulation of phage repressors of the lytic cycle. This condition not only promotes phage integration during new infections but also contributes to the persistence of existing prophages [66]. Host proteases, which rely on ATP for their proteolytic activity, serve as sensors of the cell’s energy status and are regulated by cyclic AMP [67–69]. When intracellular ATP levels are high due to an abundant energy supply, the host proteases cleave the phage repressors expressed early in the infection, thus favoring the lytic cycle [68]. Thereby, the lysogeny dependence on the host’s energetic metabolism and ATP amount is obvious. In this light, the observed pro-lysogenic role of the microRNA-size 24B_1 molecule takes on a new meaning. It seems that similar to herpesviral microRNAs, 24B_1 reprogrammes the host energy metabolism by altering the central carbon pathway and thus decreasing the cellular ATP level. The target site for the 24B_1 molecule appears to be the succinate dehydrogenase subunit gene *sdhB*, whose dysfunction may lead to disruption of the classical citric acid cycle and trigger the activation of an alternative pathway, characterized by the production of a smaller amount of ATP. In effect, the phage CI repressor is not cleaved by proteases and inhibits the phage lytic development in the bacterial cell thus maintaining the lysogeny state. This mechanism of action of the 24B_1 molecule on phage and host development seems to be highly probable in a light of the results presented in this work.

## Conclusions

In many aspects (such as size, presence of the precursor form, and occurrence of a multi-step maturation process) the phage non-coding RNA molecule, named 24B_1, resembles herpesviral microRNAs. The 24B_1 molecule plays a significant role in maintaining the prophage state and reprogramming the host’s energy metabolism. This is the first report demonstrating molecular mechanisms of functions of a microRNA-size RNA molecule of the phage origin.

## Supporting information

S1 Fig The growth kinetics of *E*. *coli* bacteria in the presence of 0.4 mM (●) and 0.8 mM (▲) concentrations of IPTG. Control *E*. *coli* bacteria (□) were cultured without the addition of IPTG.
(TIF)

S2 Fig A representative NMR spectrum for metabolic changes in *E*. *coli* bacteria induced by the overproduced 24B_1 sRNA. (TIF)

S3 Fig The visualization of the potential interaction occurring between SdhB transcript and 24B_1 molecule performed by the IntaRNA program. The 24B_1 molecule is located at the bottom, whereas the target mRNA is at the top of the illustrated interaction. The energy score was calculated by the software.
(TIF)

S1 Table Chemically synthesized RNA oligonucleotides. (DOCX)

S2 Table DNA oligonucleotides used to prepare templates for the *in vitro* transcription of the 81-nt long precursor of 24B_1 RNA and the 58-long fragment of 5ʹ-UTR of *sdhB* mRNA. (DOCX)

S1 Raw images (PDF)
