# Supplementary figures and images for: Bacteriophage-encoded 24B_1 molecule resembles herpesviral microRNAs and plays a crucial role in the development of both the virus and its host
Source: PLoS One. 2023 Dec 20;18(12):e0296038. doi: 10.1371/journal.pone.0296038 (PMC10732415; doi:10.1371/journal.pone.0296038)

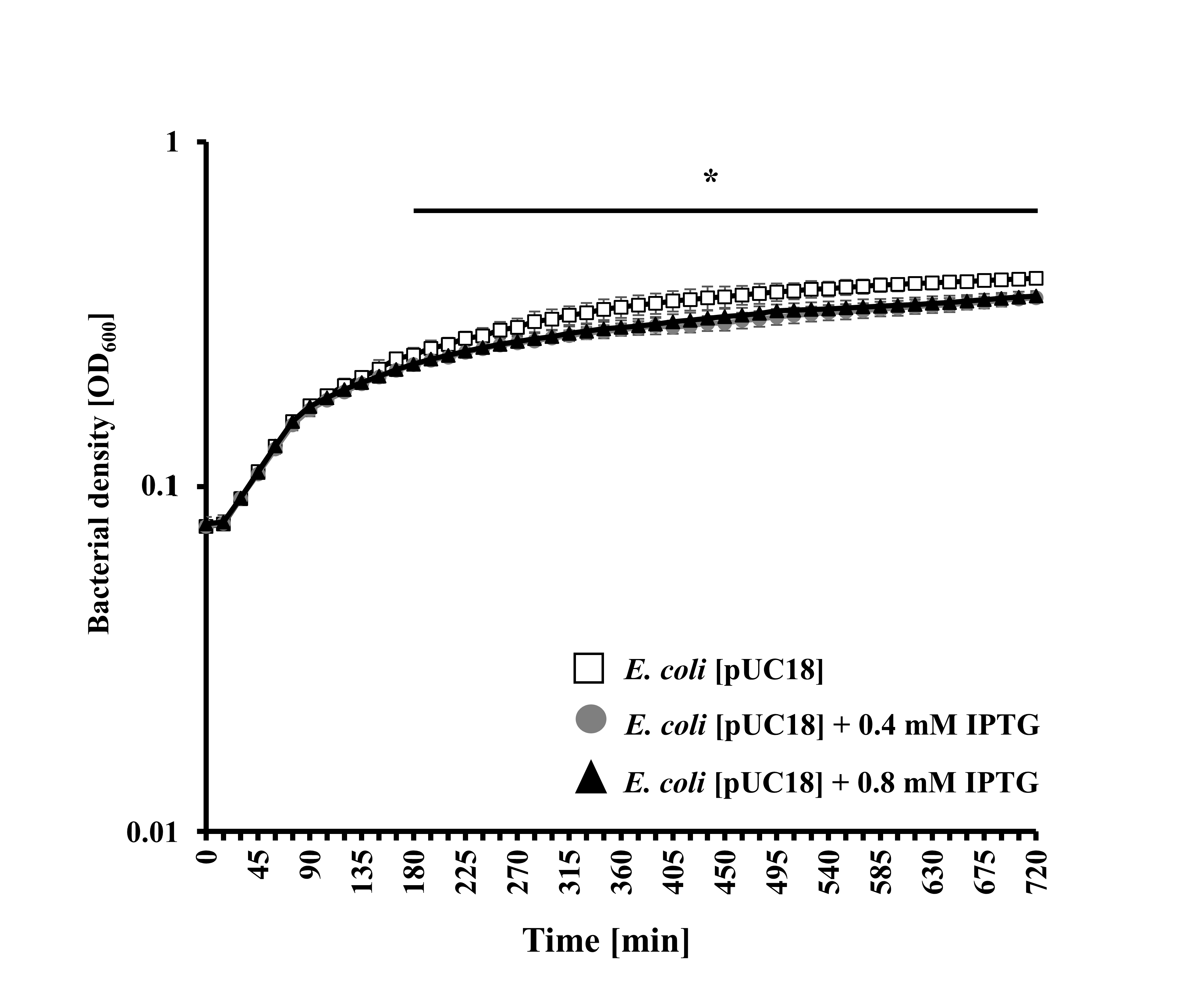

Supplement: S1 Fig — Control E. coli bacteria (□) were cultured without the addition of IPTG. (TIF) [file pone.0296038.s001.tif]

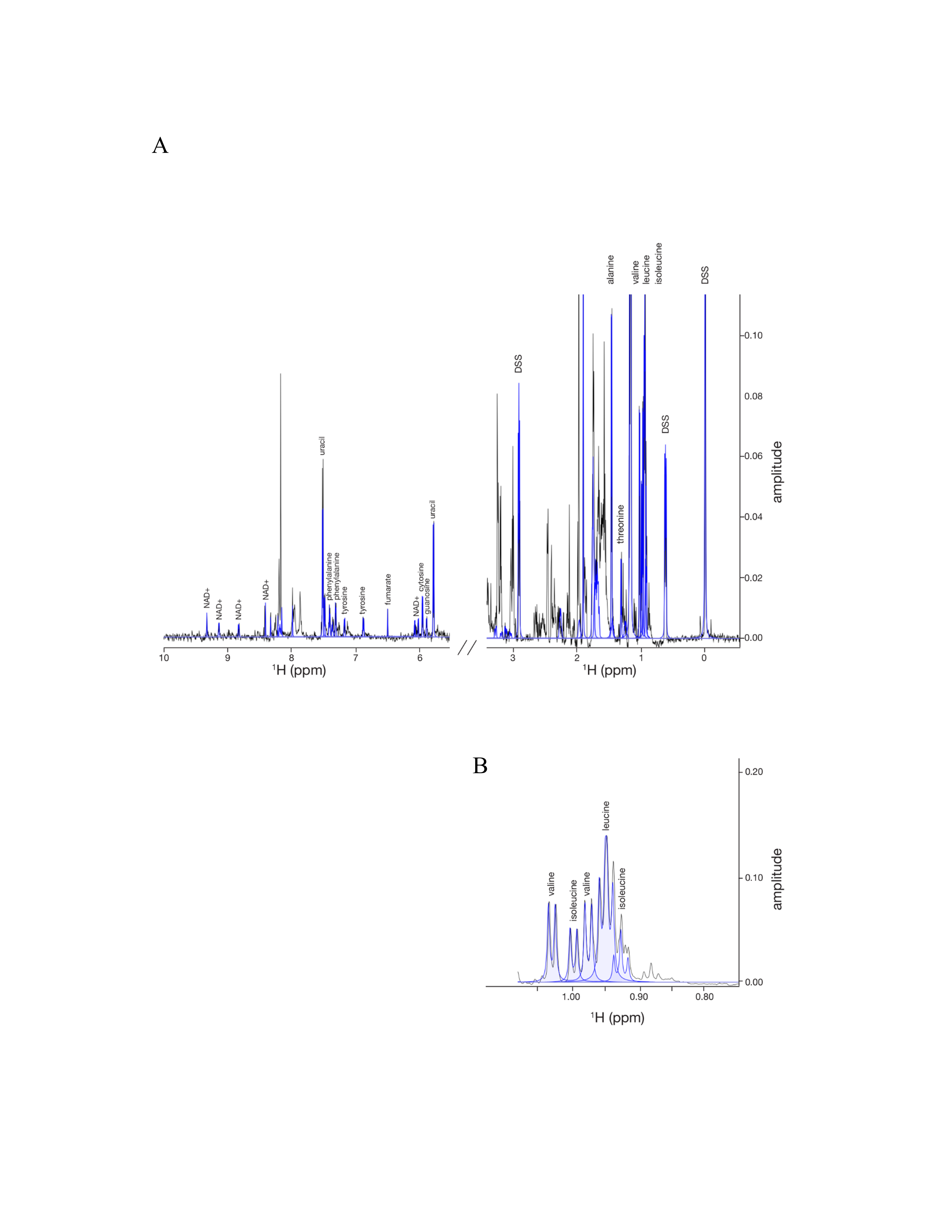

Supplement: S2 Fig — (TIF) [file pone.0296038.s002.tif]

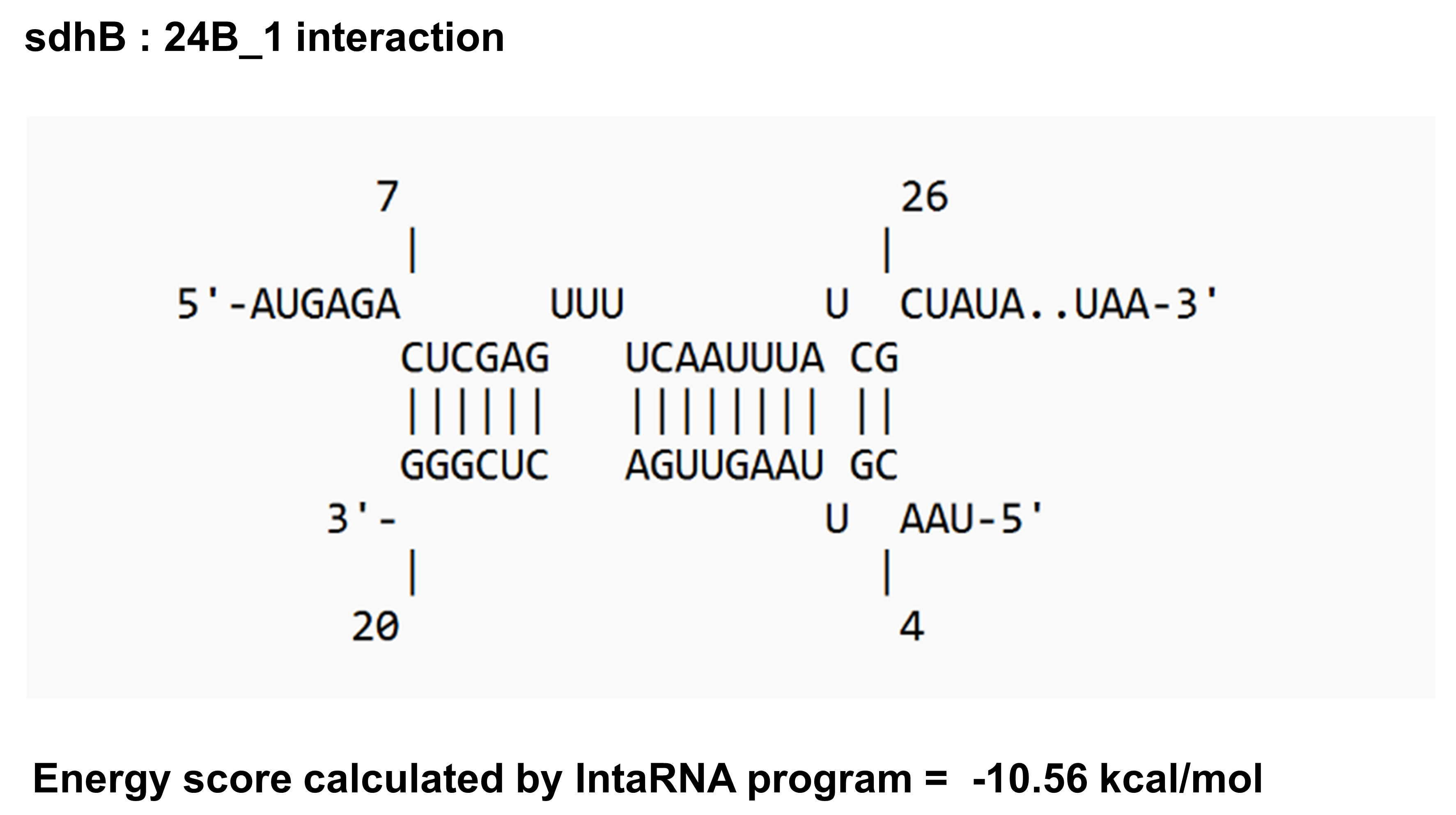

Supplement: S3 Fig — The 24B_1 molecule is located at the bottom, whereas the target mRNA is at the top of the illustrated interaction. The energy score was calculated by the software. (TIF) [file pone.0296038.s003.tif]
