# Supplementary material for: Bacteriophage-encoded 24B_1 molecule resembles herpesviral microRNAs and plays a crucial role in the development of both the virus and its host
Source: PLoS One. 2023 Dec 20;18(12):e0296038. doi: 10.1371/journal.pone.0296038 (PMC10732415; doi:10.1371/journal.pone.0296038)
Supplement: S1 Table — (DOCX) [file pone.0296038.s004.docx]

**Table S1**. Chemically synthesized RNA oligonucleotides.

| **Name** | **Sequence (5’ → 3’)** |
| --- | --- |
| 24B_1 20 | UAACGUUAAGUUGACUCGGG |
