# Supplementary material for: Bacteriophage-encoded 24B_1 molecule resembles herpesviral microRNAs and plays a crucial role in the development of both the virus and its host
Source: PLoS One. 2023 Dec 20;18(12):e0296038. doi: 10.1371/journal.pone.0296038 (PMC10732415; doi:10.1371/journal.pone.0296038)
Supplement: S2 Table — (DOCX) [file pone.0296038.s005.docx]

**Table S2.** DNA oligonucleotides used to prepare templates for the *in vitro* transcription of the 81-nt long precursor of 24B_1 RNA and the 58-long fragment of 5ʹ-UTR of *sdhB* mRNA.

| **Name** | **Sequence (5’ → 3’)** |
| --- | --- |
| 24_B1 81_F | TAATACGACTCACTATAGCTTAAGGTATGATGGTTAGGGCCTCGGGTTAACGTTAAGTTGAC TCGG |
| 24_B1 81_R | GGGTGAACCTGAAGGCCTGATGTGGAAAGGCCCCGAGTCAACTTAACGTTAACC |
| sdhB_F | TAATACGACTCACTATAGTACTTACTAATGCGGAGACAGGAAAATGAGACTCG |
| sdhB_R | TTATAGCGATAAATTGAAAACTCGAGTCTCATTTTCCTGTCTCC |
