# Supplementary material for: Bacteriophage-encoded 24B_1 molecule resembles herpesviral microRNAs and plays a crucial role in the development of both the virus and its host
Source: PLoS One. 2023 Dec 20;18(12):e0296038. doi: 10.1371/journal.pone.0296038 (PMC10732415; doi:10.1371/journal.pone.0296038)

Fig. 9 A

The image was obtained using a phosphorimager (Fujifilm FLA-5000) with ImageQuant software.

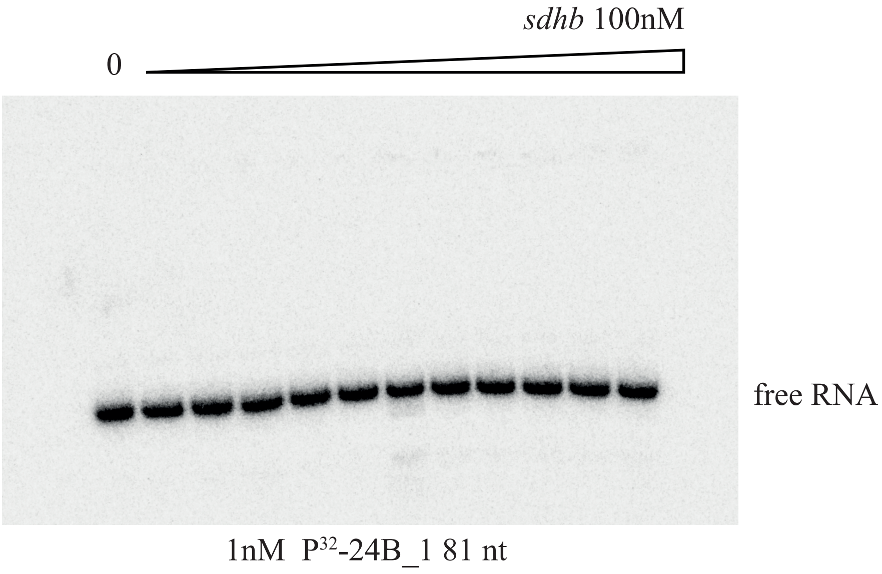

Fig. 9 B

The image was obtained using a phosphorimager (Fujifilm FLA-5000) with ImageQuant software.

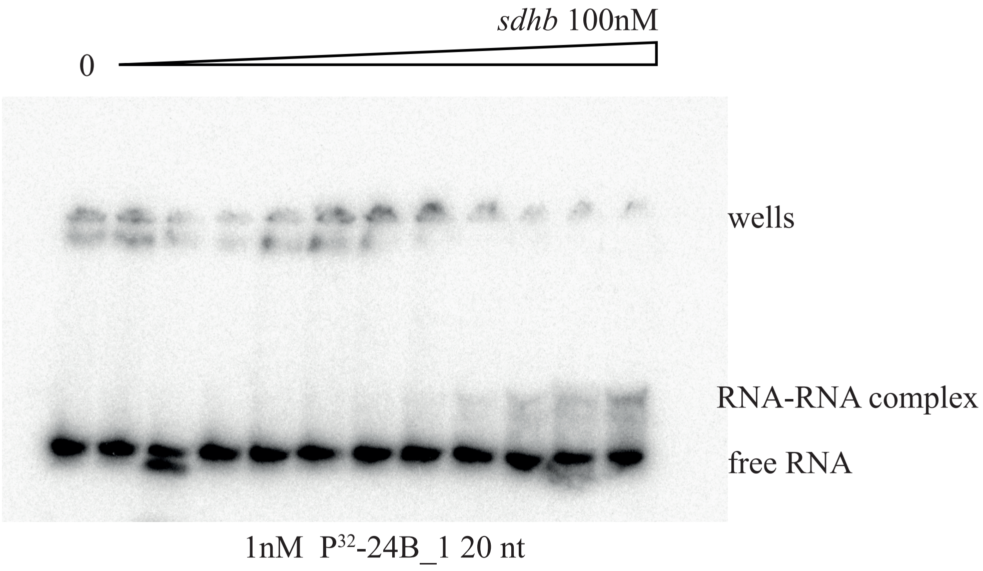

Fig. 10 A

The image was obtained using a phosphorimager (Fujifilm FLA-5000) with ImageQuant software.

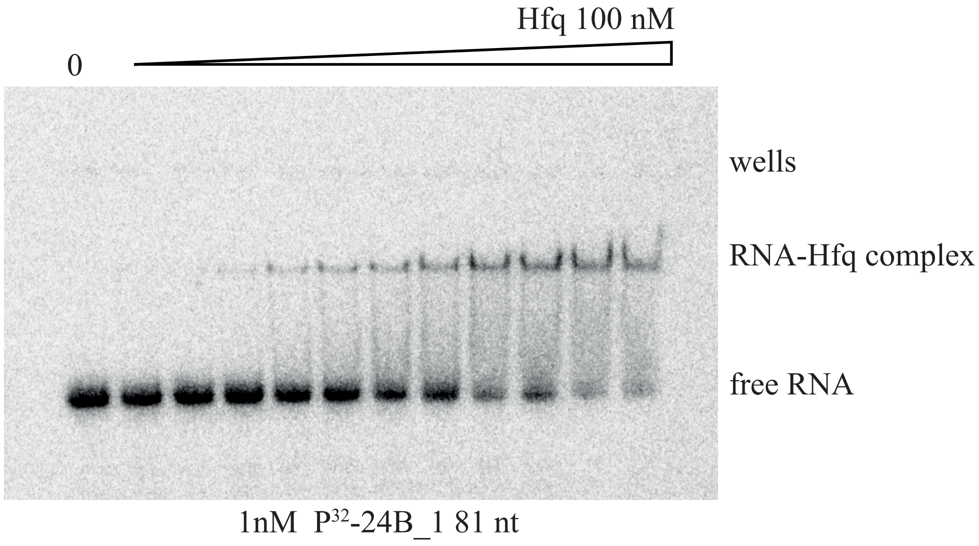

Fig. 10 B

The image was obtained using a phosphorimager (Fujifilm FLA-5000) with ImageQuant software.

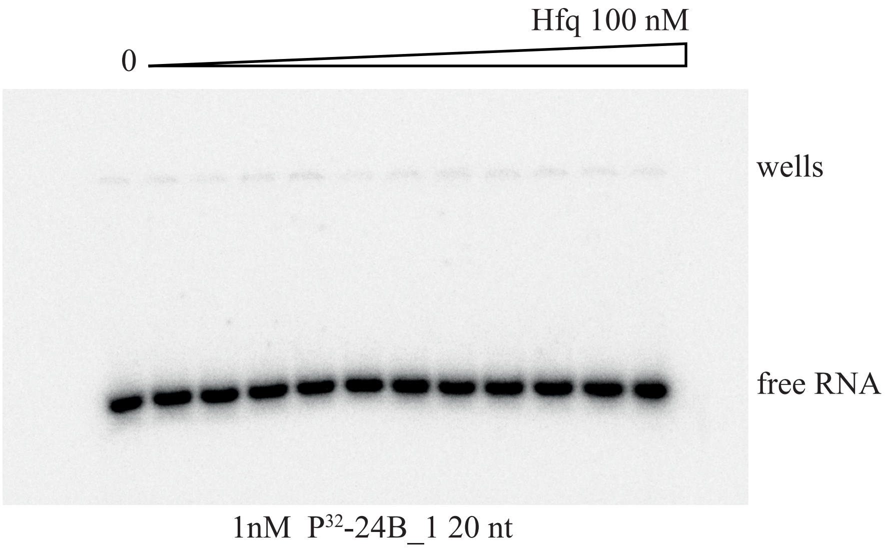

Supplement: S1 Raw images — (PDF) [file pone.0296038.s006.pdf]
